# A sandwich-like nanofibrous scaffold with macrophage phenotype transformation and myogenic differentiation for skeletal muscle regeneration

**DOI:** 10.1016/j.bioactmat.2025.05.008

**Published:** 2025-05-13

**Authors:** Shue Jin, Yongrui Cai, Yaxing Li, Jing Wen, Xiaoxue Fu, Ping Song, Pengyu Lu, Anjing Chen, Zeyu Luo, Weinan Zeng, Jidong Li, Zongke Zhou

**Affiliations:** a Department of Orthopedic Surgery and Orthopedic Research Institute, West China Hospital, Sichuan University, Chengdu, 610041, China; b Analytical & Testing Center, Sichuan University, Chengdu, 610065, China; c West China School of Medicine, Sichuan University, Chengdu, 610041, China

**Keywords:** Skeletal muscle regeneration, Sandwich-like scaffold, Hyaluronic acid, Myogenic differentiation, Macrophages phenotype transformation

## Abstract

Skeletal muscle injuries caused by trauma, infections, or sports tear are common clinical diseases. Currently, the regeneration and repair of muscle tissue, which is highly heterogeneous, remains a significant challenge. Given the anisotropic structure, high strength and tensile characteristics of skeletal muscle, this study proposes a treatment strategy for muscle injury that combines materials nano-topological cues and biochemical cues. The approach aims to facilitate muscle injury repair through the use of heterogeneous nanofibers on the surface of the sandwich-like electrospun nanofibrous scaffold and macrophage phenotype transformation. Specifically, the outer layer of the sandwich-like scaffold consists of highly aligned fibers, while the middle layer is a core-shell structured random fibers containing hyaluronic acid, and the fiber matrix is composed of optimized proportions of polycaprolactone and gelatin. Mechanical testing shows that the sandwich-like scaffold combines the excellent tensile strength of the outer aligned fibers with the larger elongation at break and suture retention strength of the inner random fibers. Cell and animal experiments confirmed that the aligned fibers in the outer layers guide the cell adhesion, cytoskeleton and nuclear remodeling, and myogenic differentiation of myoblasts, and hyaluronic acid promotes both myogenic differentiation and macrophage phenotype transformation, ultimately accelerating skeletal muscle regeneration. This sandwich-like nanofibrous scaffold provides a novel cell-free, and factor-free approach for the regeneration of skeletal muscle injuries.

## Highlights

• A cell-free and growth factor-free strategy to promote skeletal muscle regeneration.
• A sandwich-like scaffold with macrophage phenotype transformation and myogenic differentiation.
• Nano-topological cues combined with biochemical cues mediate muscle regeneration and functional recovery.

## Graphical abstract

**Figure:**
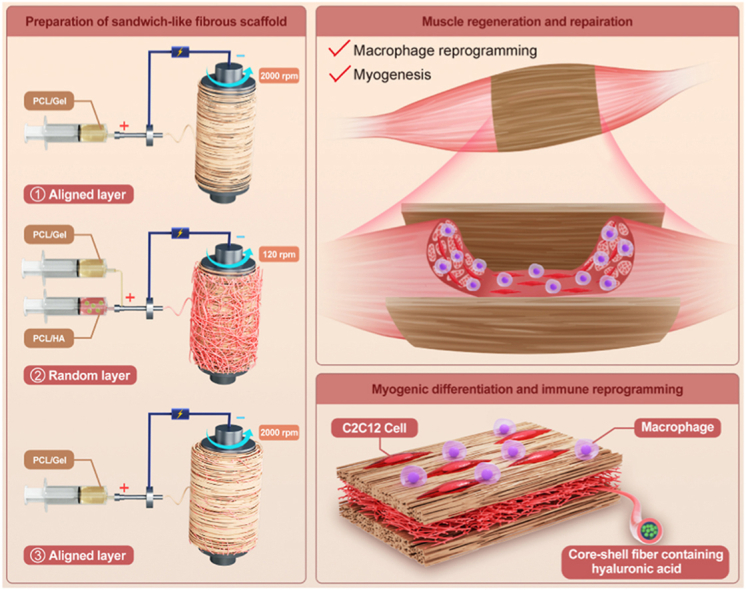

## 1 Introduction

Trauma, infections and tumor removal can cause muscle injury and even result in the loss of limb function, directly affecting patients’ health and quality of life [1]. Like other soft tissues, minor muscle injuries can often repaire themselves; however, volumetric muscle loss (VML), damage that more than 20 % of muscle mass loss, cannot regenerate due to rapid fibrosis and scar tissue formation [2,3]. Surgeons typically treat such injuries with muscle flap grafts, which require a second surgery and are limited by the availability of donor muscle tissue, and surgical interventions, which tend to lead to scarring tissue growth [4]. With the increasing prevalence of muscle injuries caused by various diseases and traumas, there is a growing need for more effective treatment strategies that promote both aesthetics and functional recovery. In recent years, with advancements in muscle tissue engineering, biomaterials-based therapeutic strategies have been iteratively updated, which are considered highly promising for transitioning from bench research to clinical practice [[5], [6], [7]].

Currently, skeletal muscle regeneration scaffolds based on cells, bioactive factors or metal ions have been widely studied [5,[8], [9], [10], [11]]. However, cells and bioactive factors are highly susceptible to deactivation due to physical shear forces and chemical environments during scaffold preparation. Additionally, while millions to billions of cells may be required to achieve a therapeutic effect, the regenerative potential of muscle is limited by the lack of autologous satellite cells and the immunosuppressive effects of allogeneic cells [12,13]. These challenges restrict the further application of cell-based therapeutic strategies. Since muscle tissue is composed of highly oriented myofibers and myocytes, there is a consensus to design and manufacture oriented scaffolds based on structural biomimetics [14,15]. Distinctive scaffolds have been designed and manufactured, although the composition and function are different, researchers are committed to preparing scaffolds with oriented heterostructure [[16], [17], [18], [19], [20]]. Electrospinning technology, a traditional nanofiber manufacturing process, is particularly useful for producing fibers with surface patterns by manipulating the receiver [21,22]. This technique offers a unique advantage in preparing oriented fibrous scaffold. In fact, electrospun oriented fibers can regulate cytoskeletal morphology and promote myogenic differentiation through mechanobiological transduction signaling axes [[23], [24], [25]]. Therefore, manipulating the surface morphology of scaffolds to regulate the behavior of myoblast-related cells and enhance muscle injury repair has become a promising alternative therapeutic strategy.

In addition to simulating the heterogeneous structure of muscle tissue, it is important to recognize that the dynamic regulation of the immune niche during skeletal muscle regeneration directly impacts the tissue repair process [26]. Immune regulation is a double-edged sword, while complete inhibition of inflammation prevents the initiation of tissue repair, persistent chronic inflammation hinders the tissue repair [27]. Even so, acute inflammation caused by scaffold implantation must be properly and promptly managed, as unaddressed inflammation can lead to fibrosis and scarring, ultimately resulting in implantation failure [28,29]. Immune cells, including macrophages, neutrophils, and dendritic cells, have been shown to create a pro-regenerative niche by secreting cytokines or directly stimulating muscle satellite cells [27,29,30]. Due to their phenotype plasticity and corresponding functional adaptability, macrophages play a dominant role in inflammatory infiltration throughout the process, from scaffold implantation to tissue regeneration completion [26]. In addition, macrophages can also secrete proteins or enzymes that directly stimulate muscle stem cell regeneration, thus improving the local muscle regeneration niche [31]. As a result, regulating macrophages phenotype has become an effective strategy for muscle immune regeneration therapy. Hyaluronic acid, a naturally occurring polymer, has been shown to regulate both inflammation and angiogenesis [32,33]. More importantly, recent study revealed that hyaluronic acid acts as a muscle generator, controlling the unique intercellular communication between muscle stem cells and immune cells during muscle injury repair, thus waking up muscle stem cells to initiate muscle repair [34]. Therefore, hyaluronic acid presents a promising candidate for regulating the muscle regeneration niche.

**Scheme 1:**
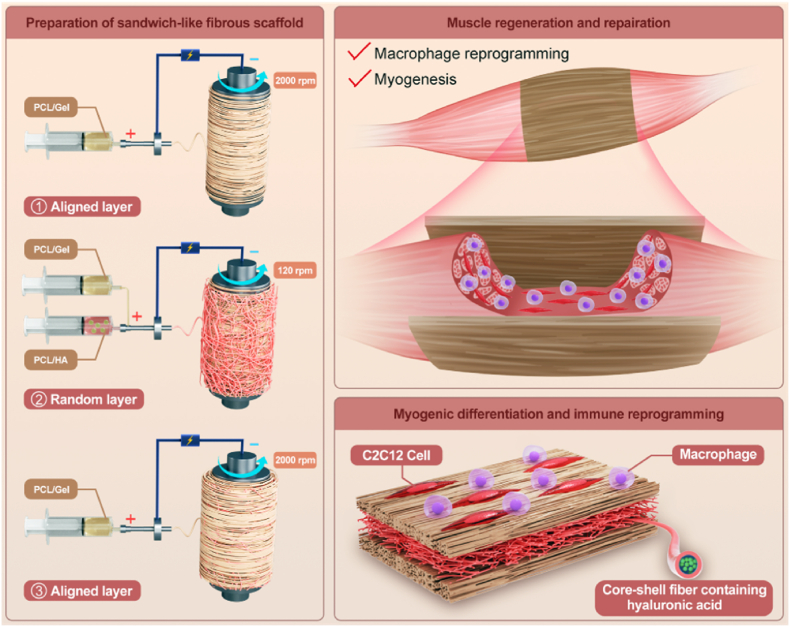
Schematic of the preparation of sandwich-like fibrous scaffold and the proposed strategy treatment for muscle injury.

Based on the above considerations, this study proposes a cell-free, factor-free treatment strategy for muscle injury, using only synthetic and natural polymers (Scheme 1). Skeletal muscle regeneration was realized from two perspectives: simulating the heterogeneous structure of muscle and macrophages phenotype transformation. Specifically, a sandwich-like, three-layer fibrous scaffold was prepared using continuous electrospinning. The upper and lower surfaces of the scaffold feature anisotropic aligned fibers, resembling the arrangement of myofibers, while the middle layer consists of random fibers with a core-shell structure containing hyaluronic acid. The aligned fiber layers are designed to regulate cytoskeleton and nuclear morphology, thereby facilitating the orientation of myocytes, myogenic differentiation, and subsequent myotube formation. The random fiber layer enhances the elongation at break and the suture retention strength of the fibrous scaffold. At the same time, the hyaluronic acid in the core-shell fibers plays a critical role in activating muscle stem cells and macrophages phenotype transformation.

In this study, we investigated the effect of pure aligned polycaprolactone (PCL) fibers on the morphology of fibroblasts and their impact on the myogenic differentiation of C2C12 cells. Then, we examined how the addition of gelatin influenced the physicochemical properties of the fibrous scaffold and optimized the ratio of gelatin to PCL. We also characterized the three-layer structure of the sandwich-like fibrous scaffold and studied its mechanical properties. The effects of different components of aligned fibrous scaffolds on cell adhesion and myogenic differentiation of C2C12 cells were evaluated. Additionally, we assessed the macrophages phenotype transformation capabilities of the sandwich-like fibrous scaffold through both *in vitro* and *in vivo* experiments. Finally, we evaluated the potential of the sandwich-like fibrous scaffold containing hyaluronic acid to promote skeletal muscle regeneration using a volumetric muscle loss (VML) model of the anterior tibial muscle, and conducted a preliminary assessment of muscle function.

## 2 Results and discussion

### 2.1 Aligned fibers reshape cell morphology and mediate myogenic differentiation

**Fig. 1:**
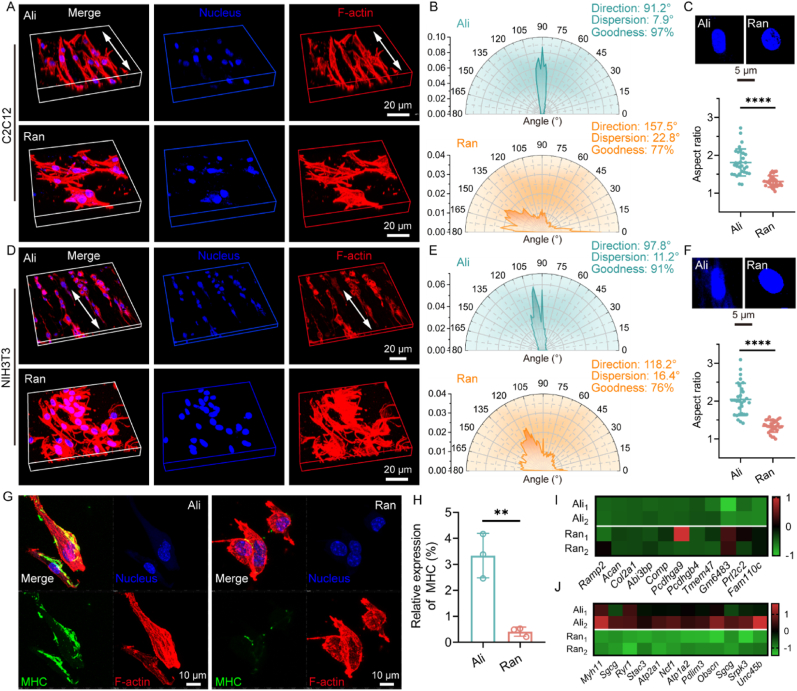
Aligned and random PCL fibers regulate cell morphology and myogenic differentiation. A, D) STROM images of cell morphology, B, E) radar maps of cytoskeleton orientation, C, F) nuclear morphology and aspect ratio of C2C12 cells and NIH3T3 cells cultured on random and aligned PCL fibrous scaffolds for 3 days. The white bidirectional arrow indicates the direction of the cytoskeleton. (n = 30; ∗∗∗∗*p* < 0.0001). G) Representative STROM images of IF staining of MHC and H) corresponding relative expression levels of C2C12 cells after 5 days of myogenic-induced culture on random and aligned PCL scaffolds (n = 3; ∗∗*p* < 0.01). Heat map of expression of I) cell morphology-related genes and J) myogenic-related genes in C2C12 cells cultured on random and aligned PCL scaffolds for 3 days.

The surface topological cues of scaffolds can directly regulate cell behavior, such as cytoskeleton remodeling, migration, spreading, proliferation and differentiation, making it possible to mediate tissue regeneration by manipulating scaffold morphology [35,36]. In this study, we aimed to mimic the anisotropic structure of myofibers by designing highly aligned electrospun fibers to guide the directional arrangement of myoblasts and ultimately promote muscle injury repair. We investigated whether aligned electrospun PCL fibers can effectively induce the orientation of fibrocytes and myocytes. Obviously, fluorescence staining showed that C2C12 cells were arranged in parallel on the aligned PCL fibers, exhibiting typical directional growth (Fig. 1A and B). The distribution of cytoskeleton was uniform. In contrast, C2C12 cells spread in different directions on the random PCL fibers, with a disorganized cytoskeleton. Moreover, C2C12 cells exhibited noticeable nuclear deformations on aligned PCL fibers, and the aspect ratio of the nuclei was significantly higher compared to those on random PCL fibers (Fig. 1C). Similarly, NIH3T3 cells displayed the same cellular morphology on aligned PCL fibers, with oriented cytoskeletal remodeling and significant nuclear deformation (Fig. 1D–F). To further investigate whether cytoskeletal remodeling and nuclear deformation induced by aligned electrospun fibers could initiate myogenesis, we performed immunofluorescence staining for myosin heavy chain (MHC). After myogenic-induced culturing, MHC expression in C2C12 cells on aligned fibers was significantly higher than that on random fibers (Fig. 1G and H). Transcriptome sequencing further revealed that on random fibers, C2C12 cells showed upregulation in genes related to cell adhesion, migration and extracellular matrix organization (Fig. 1I). In contrast, cells on aligned fibers exhibited a significant upregulation of genes involved in muscle cell differentiation and muscle contraction (Fig. 1J). These results confirmed that aligned fibers can reshape the cytoskeleton and cause nuclear deformation, ultimately eliciting distinct effect on myogenic differentiation. Previous studies have shown that nanotopographical cues, such as anisotropic surface structures, can promote myogenesis by activating Rac-related mechanotransduction signaling pathways or altering extracellular vesicle secretion [23,37]. Therefore, designing anisotropic electrospun fibers offers a potential strategy for manipulating muscle cell behavior to facilitate muscle injury repair.

### 2.2 Optimization of fiber components

**Fig. 2:**
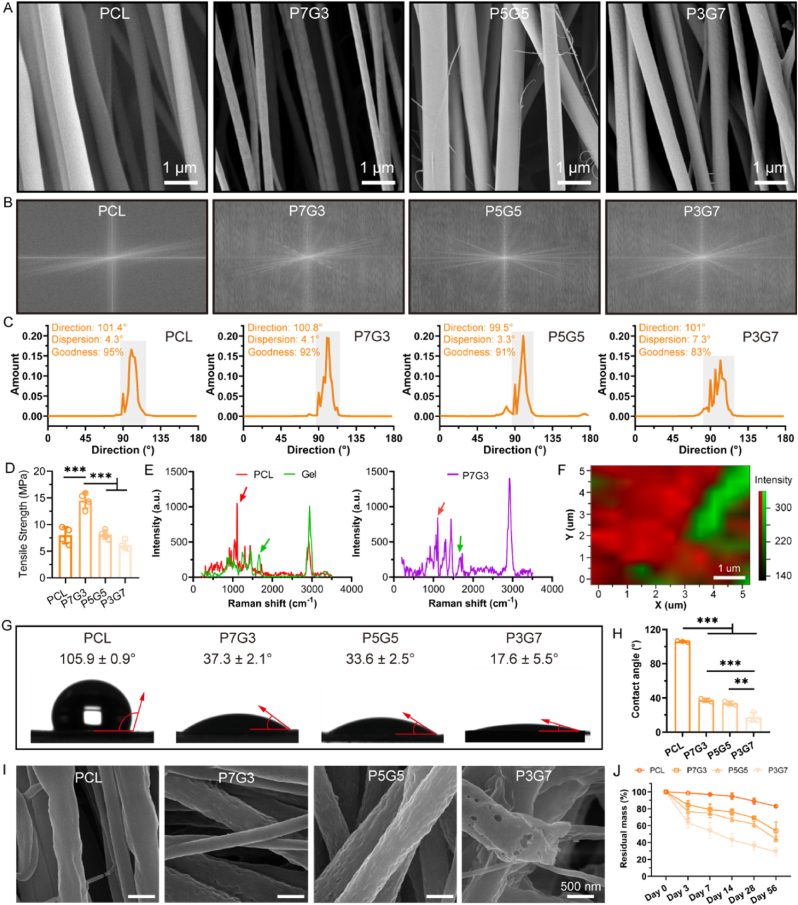
Effect of gelatin content on the properties of PCL/Gelatin fibrous scaffolds. A) Representative SEM images, B) FFT images, and C) fiber orientation distribution of PCL, P7G3, P5G5, and P3G7 scaffolds. D) Tensile strength of scaffold with different gelatin content (n = 4; ∗∗∗*p* < 0.001). E) Raman spectra of PCL, Gelatin, and P7G3. The red and green arrows indicate the characteristic peaks of PCL and gelatin at 1106 cm^−1^ and 1664 cm^−1^, respectively. F) Laser-micro confocal Raman mapping of P7G3. G) Water contact angle and H) corresponding quantitative analysis of PCL, P7G3, P5G5, and P3G7 scaffolds (n = 3; ∗∗*p* < 0.01, ∗∗∗*p* < 0.001). I) The SEM images of PCL, P7G3, P5G5, and P3G7 fibrous scaffolds after degradation for 14 days. J) The residual mass of PCL, P7G3, P5G5, and P3G7 fibrous scaffolds after degradation for different time.

PCL fibrous scaffolds have been widely used in biomedical fields due to their well-recognized biocompatibility [38,39]. However, since PCL is a semi-crystalline polymer, its degradation rate is slower compared to that of amorphous polymer [40]. This slower degradation may lead to chronic inflammation and scar formation after implantation, which hinders the rapid healing of soft tissue. Previous studies have highlighted the potential of PCL-based fibrous scaffolds for muscle regeneration. However, the surface hydrophobicity and slow degradation behavior of PCL scaffolds need to be addressed to prevent excessive fibrosis and prolonged inflammatory responses [24,38]. Gelatin, a natural polymer, has been shown to improve the physicochemical properties and degradation behavior of PCL fibrous scaffolds [41,42]. Therefore, in this study, we evaluated the effects of incorporating different proportions of gelatin into PCL fibers to optimize gelatin content. Fourier Transform Infrared Spectroscopy (FTIR) spectra confirmed the successful addition of gelatin, with the characteristic absorption peak of the amide band increasing as the gelatin content rose (Fig. S1). Scanning Electron Microscope (SEM) images showed that the inclusion of gelatin influenced the fiber morphology. The diameter of pristine PCL fibers was uniform, whereas the addition of gelatin resulted in a broader distribution of fiber diameters (Fig. 2A, Fig. S2). When the gelatin content was low (P7G3), the fiber diameter decreased to some extent. However, as the gelatin content increased further (P5G5 and P3G7), the fiber diameter did not decrease any further. This is mainly because gelatin is a polyelectrolyte rich in amino and carboxyl groups, which enhances the electrical conductivity of the electrospinning solution, promoting the formation of nanofibers with lower diameter. However, when the gelatin content is too high, it can lead to increased charge density at the syringe needle, causing an extremely unstable electrospinning jet. Fast Fourier Transform (FFT) images and the corresponding fiber alignment distribution indicated that all fibers were highly aligned, but the introduction of gelatin slightly reduced the goodness of aligned fibers (Fig. 2B and C). Mechanical testing revealed that the effect of gelatin on the tensile strength of PCL fibers initially increased and then decreased (Fig. 2D). The P7G3 scaffold exhibited the highest tensile strength of 14.51 ± 1.49 MPa. This is primarily due to the smaller fiber diameter of the P7G3 scaffold and the molecular interaction between gelatin and PCL. The distribution of gelatin within the PCL fiber matrix directly influences its surface properties and degradation performance. Confocal Raman imaging, using the characteristic Raman peaks of PCL and gelatin, showed that gelatin was distributed inhomogeneity within the PCL fiber matrix (Fig. 2E and F). Contact angle test results indicated that introducing a lower content of gelatin significantly improved the water wettability of the PCL fibrous scaffold, shifting its properties from hydrophobic to hydrophilic (Fig. 2G and H). The hydrophilicity of the implanted scaffold is crucial for cell infiltration and the subsequent inflammatory response after implantation [43]. However, excessive hydrophilicity can hinder cell spreading and migration, and may fail to trigger adequate immune response. The impact of gelatin on the degradation behavior of PCL fiber scaffolds was investigated through an *in vitro* degradation experiment. Consistent with our previous findings, gelatin induced a cavitation degradation behavior in the polymer fibers, mainly due to the significant difference in degradation rates between gelatin and PCL [44]. The high gelatin content in the P3G7 scaffold caused the premature formation of large holes in the fibers, leading to fiber fracture and, ultimately, the collapse of the fiber scaffold structure (Fig. 2I). While a faster degradation rate in scaffolds may be beneficial for skeletal muscle regeneration, rapid structural collapse not only compromises mechanical support but also disrupts local cell homeostasis. The degradation of pure PCL scaffold was very slow due to its hydrophobic surface and semi-crystalline molecular structure. In contrast, the P3G7 scaffold with high gelatin content degraded too quickly, with less than 40 % of the mass residual after 56 days (Fig. 2J). The P7G3 and P5G5 scaffolds showed similar degradation behaviors, with moderate degradation rates. Therefore, considering the fiber morphology, orientation, water wettability, mechanical properties, and degradation behavior of the fibrous scaffolds, the P7G3 scaffold—containing lower gelatin content—was selected for further preparation of the sandwich-like fibrous scaffold incorporating hyaluronic acid.

### 2.3 Morphology and structure of sandwich-like fibrous scaffold

**Fig. 3:**
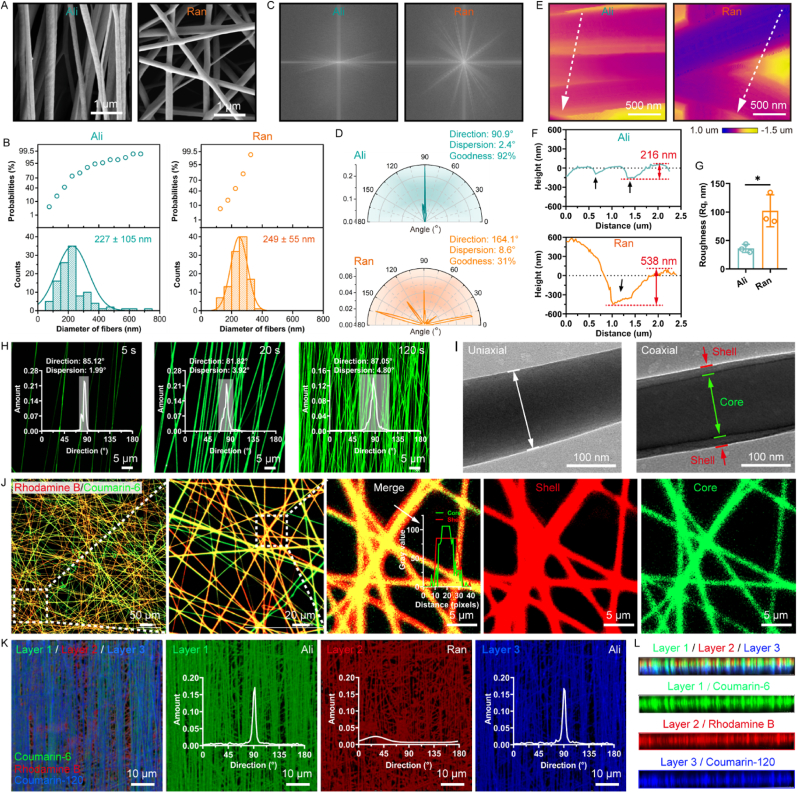
Characterization of sandwich-like PGH fibrous scaffolds. A) Representative SEM images, B) fiber diameter distribution and probabilities of aligned and random layer of PGH. C) FFT grayscale and D) fiber direction of aligned and random layer of PGH. E) AFM phase diagram of aligned and random fiber of PGH. F) Height variation of aligned and random fiber layer of PGH (obtained along the white dash arrows in (E)). G) Surface roughness of aligned and random fiber of PGH (n = 3; ∗*p* < 0.05). H) Fluorescent images of aligned fibers were collected after 5, 20, and 120 s. I) Representative TEM images of uniaxial P7G3 fibers and HA-contained coaxial fibers. J) Representative STROM images of Rhodamine and Coumarin-6-labeled coaxial random fibers. Red indicates the shell fiber labeled with Rhodamine B, green indicates the core fiber labeled with Coumarin-6. The embedded diagram is the distribution of fluorescence along the white arrow. K) Representative STROM images of Rhodamine B, Coumarin-6, and Coumarin-120-labeled three layers of PGH scaffold. Red indicates the Rhodamine B labeled random fiber layer (layer 2) of PGH, green and blue indicate Coumarin-6 and Coumarin-120 labeled aligned fiber layer (layer 1 and 3) of PGH. The embedded diagram is the direction distribution of fibers. L) Cross section of a fluorescent-labeled PGH scaffold.

The fibrous scaffolds were composed of three layers: the upper and lower layers contained aligned fibers (Ali) with a diameter of 227 ± 105 nm, while the middle layer consisted of random fibers (Ran) with a diameter of 249 ± 55 nm (Fig. 3A and B). The angle distribution of the aligned fibers was highly concentrated, whileas the angle distribution of the random fibers was more dispersed (Fig. 3C and D). Atomic force microscopy (AFM) images showed grooves with a depth of approximately 216 nm between the aligned fibers, and pores with a depth of approximately 538 nm between the random fibers (Fig. 3E and F). The difference in surface microstructure resulted in significantly higher surface roughness in the random fibers compared to the aligned fibers (Fig. 3G). The staggered arrangement of the random fibers formed bridging points that provided abundant sites for cell adhesion and spreading. In contrast, the parallel alignment of the aligned fibers facilitated cell skeleton remodeling and directional migration, as verified in Fig. 1. The structure of the fibrous scaffold was further visualized. Fluorescent images showed that the aligned fibers could be efficiently and stably collected by a high-speed rotating drum receiver, ensuring the preparation of the aligned layers in the sandwich-like fibrous scaffolds (Fig. 3H). Transmission electron microscopy (TEM) images demonstrated that the random fibers in the middle layer were coaxial core-shell fibers (Fig. 3I). Fluorescent tracking images indicated that the shell layer was made of Rhodamine-labeled PCL and gelatin (red), while the core layer consisted of coumarin-6-labeled PCL and hyaluronic acid (green), confirming the core-shell structure of the random fibers (Fig. 3J). After the implantation of fibrous scaffolds, the infiltration of tissue fluid and blood allows the core-shell structure to prevent the premature dissolution and diffusion of hyaluronic acid to some extent. Previous studies have shown that functional molecules in core-shell fibers are released through a combination of degradation and diffusion processes [45,46]. Furthermore, the sandwich-like structure of the fibrous scaffold was confirmed. Scanning electron microscopy (SEM) images of the scaffold cross-section revealed the upper, middle, and lower layers (Fig. S3). The three layers of the scaffold were labeled with Coumarin-6, Coumarin-120, and Rhodamine B, respectively. Fluorescence imaging confirmed that the fibrous scaffold exhibited a typical three-layer, sandwich-like structure (Fig. 3K and L). The fibers in the upper (Layer 1) and lower (Layer 3) layers were highly aligned, while the fibers in the middle layer (Layer 2) were randomly distributed.

### 2.4 Mechanical properties of sandwich-like fibrous scaffold

**Fig. 4:**
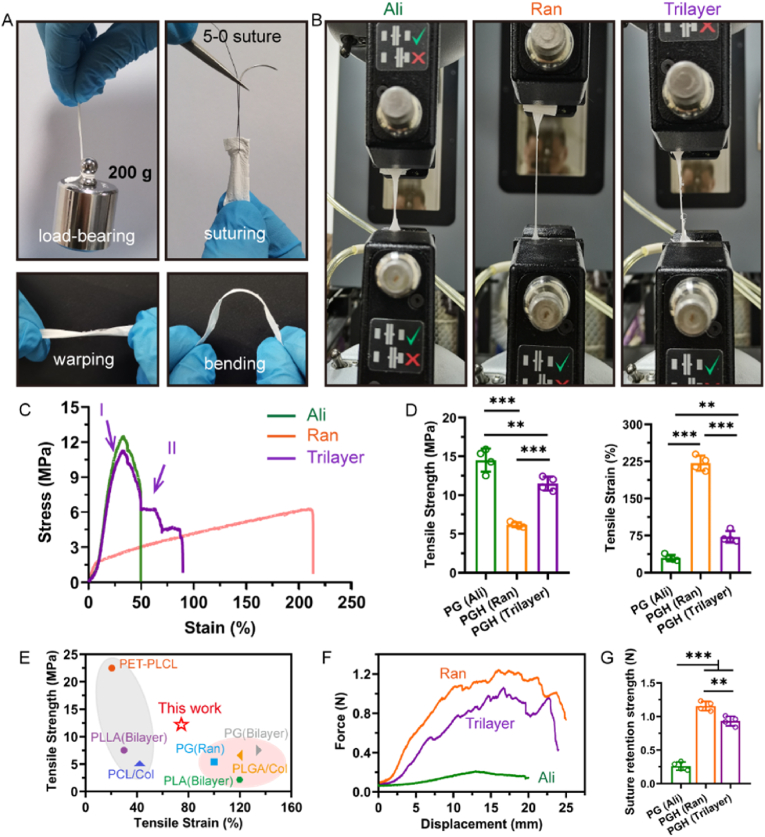
Mechanical properties of different fibrous scaffolds. A) The photo of PGH scaffold to bear load, suture cutting, warp and bend. B) Macroscopic view of the tensile process of the aligned layer, random layer, and trilayer PGH scaffolds when it was about to break. C) Tensile strain-stress curve of the aligned layer, random layer, and trilayer PGH scaffolds. D) Tensile strength and tensile strain of aligned layer (P7G3), random layer of PGH, and trilayer PGH scaffold (n = 4; ∗∗*p* < 0.01, ∗∗∗*p* < 0.001). E) Tensile strength and tensile strain comparation of the PGH scaffold with the reported electrospun scaffold based on synthetic/natural biomedical polymers. F) Typical force-displacement curves of suture retention tests of the aligned layer, random layer, and trilayer PGH scaffolds and G) corresponding suture retention strength (n = 4; ∗∗*p* < 0.01, ∗∗∗*p* < 0.001).

Skeletal muscle contracts to trigger specific movements in the body, making its mechanical properties crucial for the design of implantable skeletal muscle regeneration scaffolds. Although the sandwich-like fibrous scaffold was not expected to perfectly replicate the mechanical properties of natural muscle, it needed to be robust enough to withstand the challenges associated with implantation surgery, suturing, and subsequent muscle contractions. The plasticity of the implant is essential to meet the demands of complex clinical applications. First, the plasticity of the fibrous scaffold was tested. The results showed that the sandwich-like fibrous scaffold could support a weight of 200 g, withstand suture penetration tests, and easily bend and warp. (Fig. 4A). These findings suggest that the sandwich-like scaffold has good clinical operability. Next, the tensile strength and strain of three types of scaffolds—random fibrous, aligned fibrous, and tri-layer sandwich-like fibrous scaffolds—were evaluated using tensile tests. Macroscopic images and stress-strain curves revealed that although the aligned fibrous scaffold had strong tensile strength, its tensile strain was low (Fig. 4B and C). In contrast, the random fibrous scaffold exhibited weaker strength but larger tensile strain. The elastic deformation of Stage I of the tri-layer scaffold was similar to that of the aligned fibrous scaffold, indicating that the tensile strength of the tri-layer scaffold was primarily determined by the aligned fiber layers. In stage II, the plastic deformation showed a step-like decline, as the intermediate random fiber layer underwent macroscopic fiber alignment and microscopic orientation of molecular chains. Quantitative results showed that the tensile strength of the aligned fibrous scaffold was 14.51 ± 1.49 MPa, but its elongation at break was only 29.76 ± 6.26 % (Fig. 4D). In contrast, the random fibrous scaffold had a tensile strength of only 6.18 ± 10.32 MPa, but its elongation at break was much higher, at 221.40 ± 15.10 %. Fortunately, the tri-layer fibrous scaffold combined the advantages of both single-layer aligned (Ali) and random (Ran) fibrous scaffolds, resulting in a balanced combination of tensile strength (11.50 ± 0.89 MPa) and elongation (72.21 ± 11.81 %). Its tensile strength is much greater than that of human muscle tissue (∼1 MPa), which can meet the strength required for muscle implants in service. Compared to most electrospun scaffolds made from natural or biomedical synthetic polymers, which typically exhibit either low tensile strength or limited elongation [44,[47], [48], [49], [50], [51]], the tri-layer sandwich-like fibrous scaffold demonstrated superior mechanical performance (Fig. 4E). To simulate the clinical surgical suture process, the fibrous scaffold was stretched with a 5-0 suture to assess its suture retention strength. Anisotropic aligned fibers, while offering strong tensile strength by reducing stress concentration and defects under external forces, also have a significant drawback: they exhibit almost no suture retention strength, meaning they are prone to suture cutting [52]. As shown in Fig. 4F and G, isotropic random fibers effectively address this issue. Randomly arranged fibers resist suture cutting stress and exhibit strong suture retention strength (1.15 ± 0.07 N). The tri-layer fibrous scaffold retains the advantage of random fibers, with a suture retention strength of 0.93 ± 0.07 N, which is significantly higher than that of aligned fibers (0.26 ± 0.06 N). Overall, the sandwich-like structure and component optimization of the fibrous scaffold allow the tri-layer PGH fibrous scaffold to successfully integrate the strong tensile strength of aligned fibers with the good elongation and suture retention strength of random fibers. These outstanding mechanical properties enable the tri-layer fibrous scaffold to meet the demanding requirements of clinical procedures, suturing, and subsequent muscle contractions for skeletal muscle regeneration. It is worth noting that the mechanical properties of the scaffold during the frequent dynamic muscle contraction process are also crucial. The process of muscle contraction is often accompanied by complex mechanical environment changes. The fatigue resistance of the biomedical polymer-based sandwich-like fibrous scaffold needs to be further evaluated, and whether it can continue to be used after high frequency contraction remains to be seen. This must be considered before implementing clinical translation.

### 2.5 Adhesion and myogenic differentiation of C2C12 cells

**Fig. 5:**
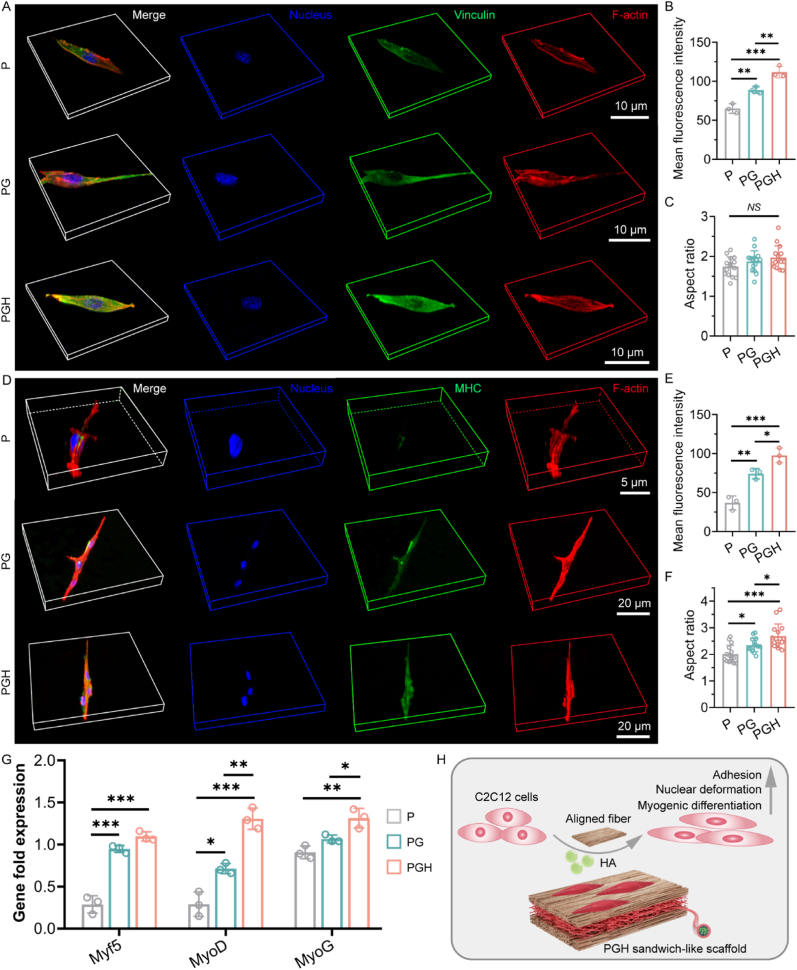
The adhesion and myogenic differentiation of C2C12 cells on the surfaces of aligned PCL scaffold, aligned PG scaffold, and the sandwich-like PGH scaffold. A) Representative STROM images of IF staining of Vinculin, B) corresponding mean fluorescence intensity, and C) aspect ratio of nucleus after 3 days of culture on P, PG, and PGH scaffolds (n = 3; ∗∗*p* < 0.01, ∗∗∗*p* < 0.001). D) Representative STROM images of IF staining of MHC, E) corresponding mean fluorescence intensity, and F) aspect ratio of nucleus after 3 days of myogenic-induced culture on P, PG, and PGH scaffolds (n = 3; ∗*p* < 0.05, ∗∗*p* < 0.01, ∗∗∗*p* < 0.001). G) qPCR analysis genes expression levels associated with myogenic differentiation in C2C12 cells after culturing on P, PG, and PGH scaffolds for 3 days (n = 3; ∗*p* < 0.05, ∗∗*p* < 0.01, ∗∗∗*p* < 0.001). H) Schematic diagram of PGH scaffold regulating C2C12 cell behavior.

Effective adhesion of cells to scaffolds is essential for scaffold-mediated cell behavior. C2C12 cells were cultured on P, PG, and PGH scaffolds for three days to investigate cell adhesion. SEM images showed that the C2C12 cells were slender and elongated on all three scaffolds. The main axis of the cytoskeleton aligned with the direction of the fibers, and the cells on the PGH and PG scaffolds exhibited more abundant pseudopods compared to those on the P scaffold (Fig. S4). Vinculin, an actin-binding protein that mediates cell-matrix and cell-cell adhesion, plays a crucial role in all stages of cell adhesion and migration. To further investigate the effects of gelatin and hyaluronic acid on cell adhesion, immunofluorescence staining for Vinculin was performed. The results clearly showed that Vinculin expression was highest in C2C12 cells on the PGH scaffold, followed by the PG scaffold, and lowest on the P scaffold (Fig. 5A and B). There was no significant difference in the aspect ratio of C2C12 cell nuclei across the three scaffolds (Fig. 5C). Therefore, the introduction of both gelatin and hyaluronic acid enhanced cell adhesion to the fibrous scaffolds without inducing nuclear remodeling. Early cell adhesion to the scaffold can directly influence subsequent cell proliferation and myogenic differentiation. CCK-8 assay results confirmed that gelatin and hyaluronic acid promoted the proliferation of C2C12 cells on the fiber scaffolds (Fig. S5). To evaluate the effect of the three scaffolds on myogenic differentiation, we examined the expression of myogenic differentiation markers in C2C12 cells after induced culturing. Immunofluorescence staining revealed that C2C12 cells were slender on the PG and PGH scaffolds and formed myotube-like structures (Fig. 5D). Semi-quantitative analysis indicated that MHC expression in C2C12 cells on the PGH scaffold was significantly higher than that on the PG and P scaffolds (Fig. 5E). (Fig. 5E). There is a new mechanism for cell-autonomous muscle repair after physiological muscle damage that does not rely on muscle stem cells but instead utilizes nuclear migration for cellular reconstruction [53]. C2C12 cells cultured on the PGH fibrous scaffold showed the largest nuclear aspect ratio (Fig. 5F), and the nuclear remodeling induced by the aligned fibers may promote muscle repair. C2C12 cells were also cultured in extracts from the three scaffolds, and the results demonstrated that hyaluronic acid significantly promoted the expression of MHC without affecting the nuclear aspect ratio (Fig. S6). Therefore, the surface pattern of aligned fibers and the presence of hyaluronic acid both facilitated myogenic differentiation of C2C12 cells. The qPCR results further indicated that after 3 days of induced culturing on PGH scaffolds, C2C12 cells showed the highest expression levels of markers for the early phase (*Myf5*) and intermediate phase (*MyoD* and *MyoG*) of myogenic differentiation, while expression levels were lowest on the P scaffold (Fig. 5G). Gelatin primarily promoted cell adhesion, proliferation, and myogenic differentiation by altering the surface properties of the fibrous scaffold, specifically its hydrophilicity. In contrast, hyaluronic acid directly initiated myogenic differentiation of C2C12 cells. As shown in Fig. 5H, the PGH scaffold promoted cell adhesion and regulated cytoskeletal and nuclear remodeling through the aligned fibers of the outer layer, thereby mediating myogenic differentiation. Importantly, the hyaluronic acid in the coaxial fibers of the intermediate layer directly promoted myogenic differentiation. Overall, the PGH scaffold effectively mediates myogenic differentiation over time, which is crucial for initiating skeletal muscle regeneration and repair.

### 2.6 Evaluation of macrophages phenotype transformation *in vivo* and *in vitro*

**Fig. 6:**
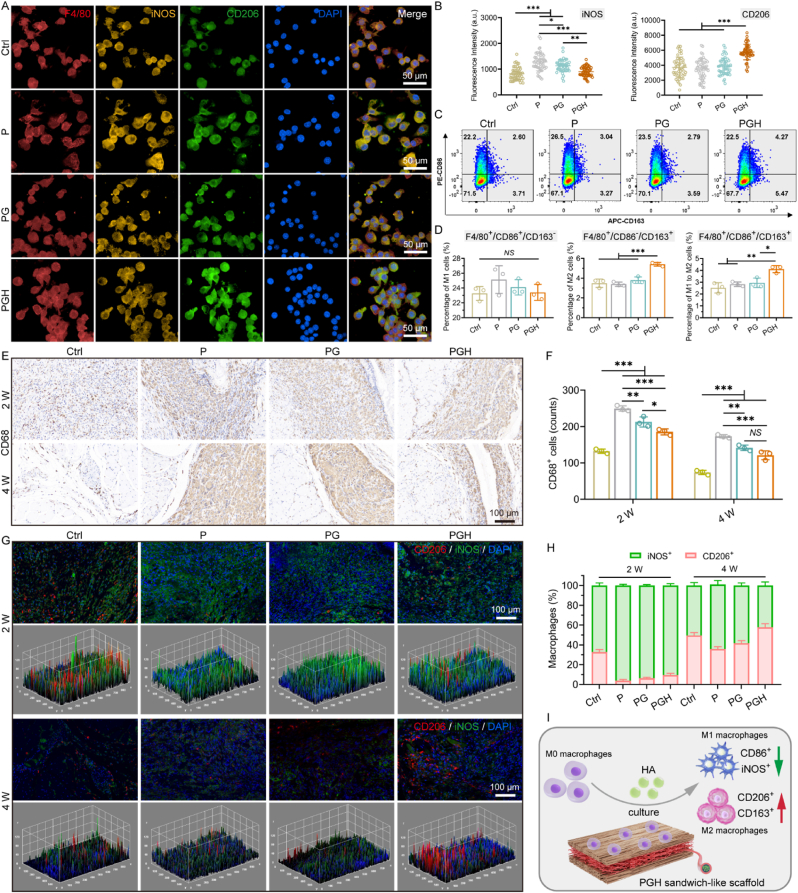
Evaluation of macrophages phenotype transformation mediated by fibrous scaffolds *in vitro* and *in vivo*. A) Representative fluorescence images and B) corresponding fluorescence intensity analysis of iNOS/CD206 expression in RAW264.7 cells after culturing for 24 h (n = 30; ∗*p* < 0.05, ∗∗*p* < 0.01, ∗∗∗*p* < 0.001). C) Phenotype of RAW264.7 cells cultured on different scaffolds for 24 h were detected by flow cytometry. D) Quantification of the percentage of M1 macrophage (F4/80^+^/CD86^+^/CD163^-^), M2 macrophage (F4/80^+^/CD86^-^/CD163^+^), and M1 to M2 (F4/80^+^/CD86^+^/CD163^+^) macrophage (n = 3; ∗*p* < 0.05, ∗∗*p* < 0.01, ∗∗∗*p* < 0.001). E) Representative images of IHC staining for CD68^+^ monocyte/macrophages after 2 weeks and 4 weeks of TA muscle injury model. F) Semi-quantitative analysis of CD68^+^ monocyte/macrophages around scaffolds (n = 3; ∗*p* < 0.05, ∗∗*p* < 0.01, ∗∗∗*p* < 0.001). G) Representative fluorescence images and corresponding 3D surface plot of M1 (iNOS^+^, green) and M2 (CD206^+^, red) macrophages recruited after 2 weeks and 4 weeks of TA muscle injury model. H) Quantified the number of M1 (iNOS^+^) and M2 (CD206^+^) macrophages and calculated the corresponding percentage. I) Schematic diagram of the PGH scaffold regulate macrophages phenotype transformation.

Macrophages are not essential for the activation of muscle satellite cells (MuSCs); however, they play a stage-dependent role in the subsequent proliferation and differentiation of MuSCs, which is crucial for effective muscle regeneration [54,55]. The beneficial role of macrophages phenotype transformation in inhibiting fibrosis and regulating inflammation has also been well-verified [56,57]. Therefore, given the central role of immune modulatory interventions in tissue repair, macrophages phenotype transformation may be a promising strategy to enhance skeletal muscle regeneration. The effect of fibrous scaffolds on macrophages phenotype transformation *in vitro* was evaluated by assessing the polarization phenotypic transformation of RAW264.7 cells. Fluorescence staining revealed that the expression of iNOS in the PGH group was the lowest, while the expression of CD206 in the PGH group was significantly higher than in the other groups (Fig. 6A and B). Flow cytometry results showed no significant difference in the proportion of pro-inflammatory M1 macrophages (F4/80^+^/CD86^+^/CD163^-^) in RAW264.7 cells cultured on P, PG, and PGH scaffolds for 24 h. However, the proportion of anti-inflammatory M2 macrophages (F4/80^+^/CD86^-^/CD163^+^) was greater in the PGH group (Fig. 6C and D). Moreover, the PGH group had a higher percentage of M1-to-M2 macrophage polarization (F4/80^+^/CD86^+^/CD163^+^), and this type of macrophage could undergo phenotypic transformation to regulate the local immune environment, playing a critical role in the pro-regeneration process [58]. Therefore, the *in vitro* results suggest that PGH fibrous scaffolds containing hyaluronic acid promoted macrophages phenotype transformation by reversing the polarization of dual-identity macrophages to the M2 phenotype, rather than directly reducing M1 macrophage polarization.

Considering that the polarization of RAW264.7 cells *in vitro* occurs in a simplified cellular niche, which cannot accurately replicate the complex local immune microenvironment *in vivo*, scaffold-mediated macrophages phenotype transformation at the implantation site was further investigated [59]. The macrophages leading immune response following the implantation of three different scaffolds was assessed using volume muscle loss (VML) model. Immunohistochemical staining showed that the P scaffold led to a dramatic infiltration of monocytes/macrophages expressing CD68, while the PGH scaffold was surrounded by the fewest CD68^+^ cells (Fig. 6E and F), indicating that the addition of gelatin and hyaluronic acid significantly reduced the inflammatory response caused by scaffold implantation. To further identify the subtypes of CD68^+^ macrophages, immunofluorescence staining was performed (Fig. 6G and H). Two weeks after implantation, most of the macrophages surrounding the fibrous scaffold were pro-inflammatory cells expressing high levels of iNOS. After 4 weeks, the number of regenerative CD206^+^ macrophages increased significantly, with the PGH group showing the highest proportion of CD206^+^ macrophages. *In vivo* results confirmed that the PGH fibrous scaffold can reverse macrophage polarization and create an adjustable immune niche that supports skeletal muscle regeneration. This favorable immune regulation can block inflammatory signaling, reduce excessive fibrosis, and regulate muscle stem cell activity, ultimately promoting skeletal muscle regeneration [29,56,60]. In summary, as shown in Fig. 6I, the PGH scaffold promotes the activation of M0 macrophages into regenerative M2 macrophages and reduces the activation of pro-inflammatory M1 macrophages. This is due to the hyaluronic acid content and the hydrophilic surface properties of the scaffold. The macrophages phenotype transformation potential of the PGH scaffold provides a favorable immune niche for skeletal muscle regeneration.

### 2.7 Skeletal muscle regeneration mediated by sandwich-like fibrous scaffold

**Fig. 7:**
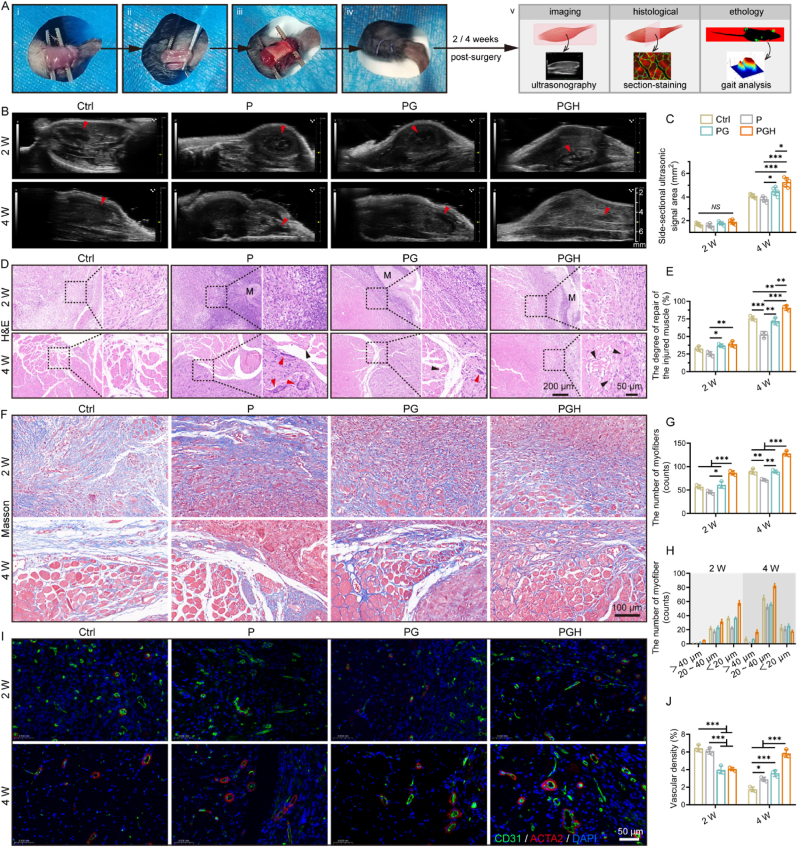
The regulation of fibrous scaffolds on skeletal muscle regeneration was evaluated by TA muscle injury model. A) Photos of TA muscle injury model and methods of postoperative detection. B) Representative ultrasound images (longitudinal section) collected two and four weeks after surgery. The red arrow indicates the defect area. C) Semi-quantification of side-sectional ultrasonic signal in defect area (n = 5; ∗*p* < 0.05, ∗∗*p* < 0.01, ∗∗∗*p* < 0.001). D) Representative H&E staining images of the scaffold implantation after two and four weeks. The right panels are the magnified images of the dashed box in the left panel. Black arrows indicate mature blood vessels and the red arrows indicate multinucleated foreign body giant cells. The capital M indicates scaffolds. E) Semi-quantified the degree of repair of the injured muscle according to H&E results (n = 3; ∗*p* < 0.05, ∗∗*p* < 0.01, ∗∗∗*p* < 0.001). F) Representative Masson staining images of the scaffold implantation after two and four weeks. G) Quantification of the number of newly formed myofibers (n = 3; ∗*p* < 0.05, ∗∗*p* < 0.01, ∗∗∗*p* < 0.001). H) Quantified the number of newly formed myofibers in different diameter. I) Representative fluorescence images of blood vessels marked by CD31 (red) and ACTA2 (green) after two and four weeks of implantation. J) Quantified the vascular density according to the positive expression of CD31and ACTA2 (n = 3; ∗*p* < 0.05, ∗∗∗*p* < 0.001).

The effect of different fibrous scaffolds on muscle injury repair was evaluated using the volumetric muscle loss (VML) model in mice, focusing on the tibialis anterior (TA) muscle. The surgical process and the corresponding postoperative detection methods are shown in Fig. 7A. Nondestructive testing of muscle repair process was performed using ultrasound imaging. The muscle tissue displayed a uniform linear structure under ultrasonic imaging, with the hyperechoic perimysium surrounding hypoechoic muscle fibers. Two weeks after surgery, the ultrasound images revealed only a small amount of high-echo tissue, with low-echo linear structures in the defect area for all groups (Fig. 7B). However, four weeks after repair, the linear structures increased significantly in all groups, indicating the formation of new muscle fiber bundles. Semi-quantitative analysis showed that the PGH group exhibited the largest area of strong ultrasonic signals in the defect region after 4 weeks of implantation (Fig. 7C).

Histological analysis was conducted to further investigate how the three scaffolds guide skeletal muscle regeneration. Two weeks after surgery, a dense blue-purple stained nucleus was observed around the P scaffold, indicating significant inflammatory cell infiltration. In contrast, the inflammatory response around the PG and PGH scaffolds was much milder (Fig. 7D). The P scaffold group showed only a small number of newly formed muscle fibers, whereas muscle fiber formation was more pronounced in the PG and PGH groups. After four weeks of implantation, inflammation was significantly reduced in all groups, and a large number of muscle fibers had formed. Additionally, mature neovascularization networks were observed in the PGH group, while the P group exhibited only a few mature blood vessels, along with more multinucleated foreign body giant cells. To evaluate the degree of muscle repair, the proportion of new muscle fibers was analyzed from H&E staining images. The results indicated that the muscle repair was most advanced in the PGH group, both two and four weeks after implantation (Fig. 7E). Masson staining revealed an abundance of blue-stained collagen fibers in all groups two weeks after surgery, but these were less abundant four weeks post-surgery (Fig. 7F). In contrast, red-stained muscle fibers increased significantly four weeks after surgery. Quantitative analysis showed that the number of new muscle fibers was lowest in the P group and highest in the PGH group (Fig. 7G). Notably, compared to the P and PG groups, the PGH group had more budding muscle fibers (<20 μm in diameter) two weeks after implantation and more mature (>40 μm in diameter) and growing (20–40 μm in diameter) muscle fibers four weeks after implantation (Fig. 7H). Ultrasound imaging and histological analysis demonstrated that the PGH scaffold initiate muscle repair earlier and continuously promoted the growth, development, and maturation of muscle fibers during the later stages of regeneration. In addition, we preliminarily evaluated the degradation behavior of the different scaffolds *in vivo* through macroscopic photographs taken at sample collection. As shown in Fig. S7, two weeks after implantation, no obvious structural damage was observed in the three fiber scaffolds, while four weeks after implantation, the PG and PGH fibrous scaffolds showed obvious rupture and deformation, while the structure of P scaffold remained intact, indicating that the PG and PGH scaffolds degraded to varying degrees. Fortunately, the three scaffolds-mediated muscle regeneration processes did not show obvious redness, swelling and abscess caused by excessive inflammatory response. Therefore, the degradation performance of the sandwich-like scaffold was able to maintain muscle regeneration without significant structural collapse and biotoxicity for at least 4 weeks after surgery.

Platelet-derived chemokines and vascular formation are critical for regulating early inflammation and promoting later muscle fiber growth in the treatment of muscle injuries [61,62]. H&E staining images clearly showed that neovascularization networks formed in all groups, but there were differences in blood vessel density and maturity. To further assess neovascularization during skeletal muscle regeneration, immunofluorescence staining for vascular system markers (CD31 and ACTA2) was performed (Fig. 7I and J). Two weeks after surgery, abundant tumor-like capillaries appeared in all groups. These capillaries mainly delivered cytokines and played a role in regulating the immune niche during the early stages of tissue repair, but eventually, they degenerated and did not form functional vasculature. In the PG and PGH groups, a small number of mature blood vessels, marked by positive expression of CD31 and ACTA2, were observed. After four weeks, some capillaries gradually developed into mature blood vessels. Quantitative analysis revealed that the density of mature vasculature was highest in the PGH group. The mature blood vessels provided the necessary cytokines and nutrient channels required for skeletal muscle regeneration.

**Fig. 8:**
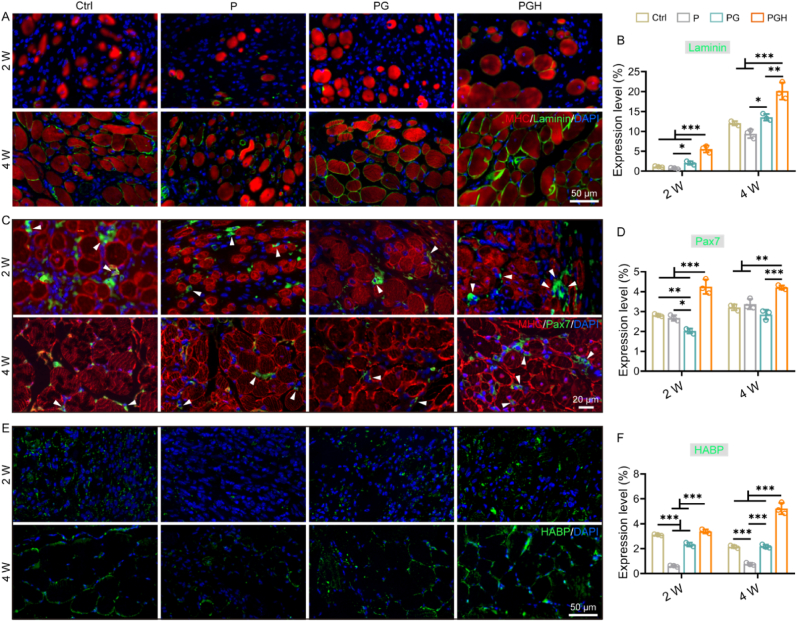
IF staining analysis of proteins associated with skeletal muscle regeneration. A) Representative fluorescence images of IF staining of MHC (red) and Laminin (green) after two and four weeks of implantation. B) Semi-quantified the positive expression level of Laminin (n = 3; ∗*p* < 0.05, ∗∗*p* < 0.01, ∗∗∗*p* < 0.001). C) Representative fluorescence images of IF staining of MHC (red) and Pax7 (green) after two and four weeks of implantation. B) Semi-quantified the positive expression level of Pax7, which is a marker of myosatellite cells (n = 3; ∗*p* < 0.05, ∗∗*p* < 0.01, ∗∗∗*p* < 0.001). E) Representative fluorescence images of IF staining of HABP (green) after two and four weeks of implantation. B) Semi-quantified the positive expression level of HABP (n = 3; ∗∗∗*p* < 0.001).

After ultrasonic imaging and histological evaluation of the newly formed muscle, the expression of specific markers related to skeletal muscle regeneration was further assessed using immunofluorescence. Staining for the basal lamina component (Laminin) was employed to outline the myofiber boundaries and reflect the maturity of muscle fibers. Myosin heavy chains (MHC) were used to identify regenerating fibers. Two weeks after implantation, loosely arranged new muscle fibers were observed in all groups, showing high expression of MHC but low expression of Laminin (Fig. 8A and B). Muscle fiber size was significantly larger in the PGH group. Four weeks post-implantation, the injury site treated with P scaffold exhibited limited signs of regenerating muscle fibers, while the injury sites treated with PG and PGH scaffolds showed well-arranged, multiple regenerating muscle fibers with high expression of both MHC and Laminin. Satellite cells, a canonical muscle stem cells that dominates muscle growth and regeneration after injury, are highly expressive of Pax7, an essential transcription factor for satellite cell maintenance [63,64]. Immunofluorescent staining results revealed that the PGH group had more Pax7^+^ satellite cells at both two weeks and four weeks post-surgery. This suggests that the PGH scaffold activated more resting satellite cells, prompting them to rapidly proliferate into myoblasts, which ultimately differentiate and fuse to initiate skeletal muscle regeneration (Fig. 8C and D). The activation of satellite cells is closely related to the regenerative niche of injured muscle and the hyaluronic acid that surrounds satellite cells [34]. We have demonstrated that PGH scaffolds can initiate macrophages phenotype transformation, enabling macrophages to switch to a pro-regenerative M2 phenotype. The biomechanical and bioactive roles of endogenous hyaluronic acid in skeletal muscle tissue regeneration and repair have also been demonstrated [34,65]. To further investigate the role of exogenous hyaluronic acid from PGH scaffolds in the skeletal muscle regeneration niche, we measured the expression of hyaluronic acid-binding protein (HABP) by immunofluorescent staining (Fig. 8E and F). Among all the implanted scaffold groups, the PGH group exhibited the highest level of HABP expression, indicating that exogenous hyaluronic acid in the PGH scaffold binds to HABP in the tissues. This interaction likely contributes to the regulation of satellite cell activation and the regeneration niche. Histological results showed that the muscle defects repaired by PGH scaffolds were more similar to normal muscles (Fig. S8). Overall, the PGH scaffold triggered a beneficial cascade response following implantation, not only regulating local macrophages microenvironment but also activating satellite cells, ultimately creating a supportive niche for skeletal muscle regeneration.

### 2.8 Transcriptome sequencing analysis

**Fig. 9:**
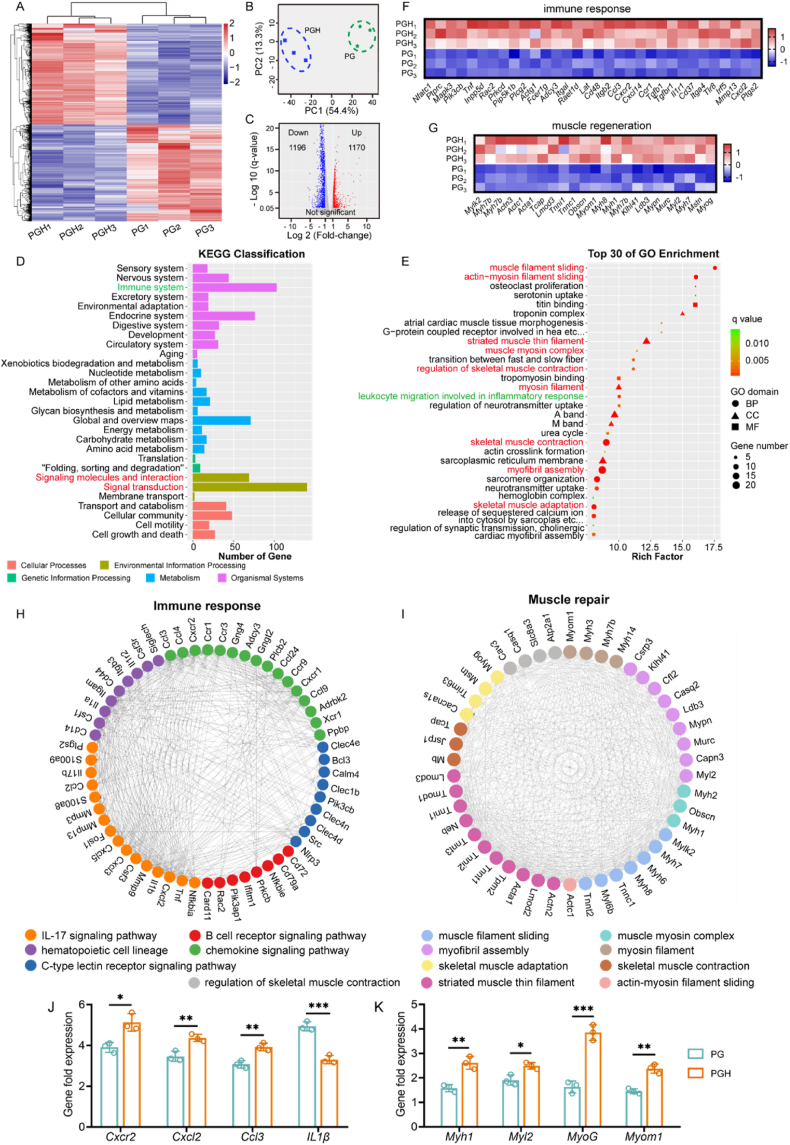
Transcriptome analysis of skeletal muscle regeneration mediated by PG and PGH scaffolds. A) Heatmap hierarchical cluster analysis of differentially expressed genes in TA muscle injury model between PG and PGH scaffolds. B) Principal component analysis of gene expression of PG and PGH group. C) Volcano map of differentially expressed genes for PGH vs PG. D) KEGG classification and E) GO enrichment of differentially expressed genes for PGH vs PG. Heatmap analysis of differentially expressed genes associated with F) immune response and G) muscle regeneration. Protein-protein interaction network analysis of up-regulated differentially expressed genes associated with H) immune response and I) muscle repair for PGH vs PG. qPCR analysis of representative genes expression levels associated with J) immune response and K) muscle repair for PGH and PG scaffolds (n = 3; ∗*p* < 0.05, ∗∗*p* < 0.01, ∗∗∗*p* < 0.001).

In addition to ultrasound imaging and histological analysis, we further explored genes associated with fibrous scaffold-mediated skeletal muscle regeneration through transcriptome sequencing. Hierarchical clustering of differentially expressed genes revealed that the PGH group clustered separately from the PG group (Fig. 9A). Principal component analysis (PCA) showed distinct clustering within the groups, with PC1 explaining 54.4 % and PC2 explaining 13.3 % of the variance, indicating significant differences between the PGH and PG groups (Fig. 9B). The volcano plot of differentially expressed genes showed 1170 upregulated genes and 1196 downregulated genes in the PGH vs. PG comparison, suggesting a positive or negative correlation between their expression and muscle injury repair (Fig. 9C). KEGG pathway analysis indicated that, compared to the PG scaffold-treated injury, the PGH scaffold-treated injury was significantly enriched in pathways related to the immune system, endocrine system, signaling molecules and interactions, and signal transduction (Fig. 9D). Furthermore, GO enrichment analysis indicated that VML model treated with PGH group had more upregulated genes concerning with muscle filament sliding, actin-myosin filament sliding, striated muscle thin filament, muscle myosin complex, regulation of skeletal muscle contraction, myosin filament, skeletal muscle contraction, myofibril assembly, skeletal muscle adaption, and leukocyte migration involved in inflammatory response (Fig. 9E). These significantly upregulated GO terms were primarily associated with muscle function and the inflammatory response. Among the differentially expressed genes and upregulated terms, we focused specifically on immune response and skeletal muscle regeneration. We identified 32 representative genes, such as *Cxcr2*, *Cxcl2*, *Rac2*, and *Ccl3*, involving in immunoregulation, inflammatory response, and chemokine recruitment, which were highly expressed in muscle injury treated with the PGH scaffold (Fig. 9F). They regulate cytokine expression, which plays a crucial role in the immune niche of skeletal muscle regeneration, mediated by monocytes, macrophages, and neutrophils. Additionally, many genes related to skeletal muscle regeneration, such as *Myom1*, *Myh1*, *Myl2*, and *Myog*, also showed similarly high expression in the PGH group (Fig. 9G). Furthermore, the protein-protein interaction network revealed that muscle injury treated with the PGH scaffold not only alters the expression of individual genes but also changes the co-expression patterns of these genes related to immune response, including IL-17 signaling pathway, B cell receptor signaling pathway, hematopoietic cell lineage, chemokine signaling pathway, and C-type lectin receptor signaling pathway) and skeletal muscle regeneration (including muscle filament sliding, muscle myosin complex, myosin filament, myofibril assembly, skeletal muscle adaption, skeletal muscle contraction, striated muscle thin filament, actin-myosin filament sliding, and regulation of skeletal muscle contraction (Fig. 9H and I). As shown in Fig. 9J and K, the expression of representative key genes was further verified. Therefore, the transcriptomic analysis suggests that the PGH scaffold plays a more significant role than the PG scaffold in the positive regulation of genes related to immune response and skeletal muscle regeneration.

### 2.9 Muscle function assessment

**Fig. 10:**
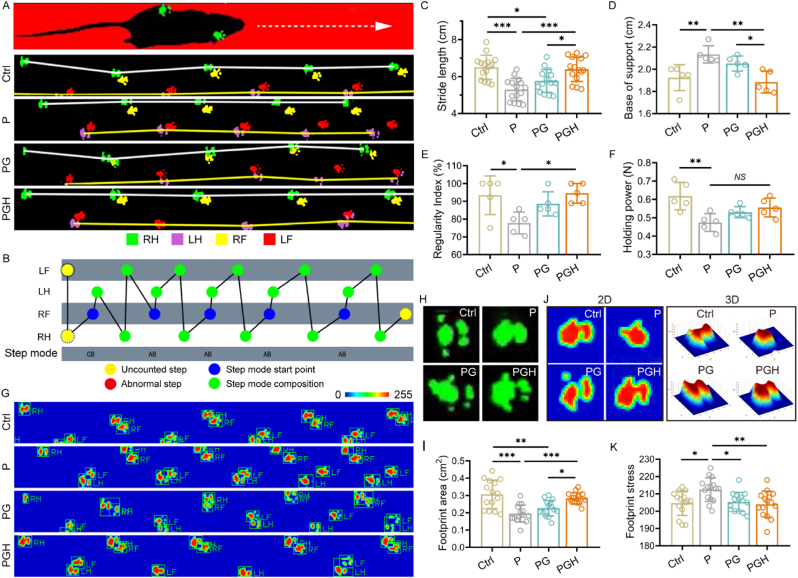
The recovery of TA muscle function was assessed by gait analysis and grip test at 4 weeks after surgery. A) Representative marching graph of mice in each group during the gait test. B) Representative step-sequence chart of mice in PGH group. C) Quantitative analysis of the stride length (n = 15; ∗*p* < 0.05, ∗∗∗*p* < 0.001). D, E) Quantitative analysis of gait indices BOS (Base of support) and RI (Regularity index) reflecting motor function (n = 3; ∗*p* < 0.05, ∗∗*p* < 0.01). F) Quantitative analysis of the holding power of hind limb via grip test (n = 3; ∗∗*p* < 0.01). G) Footprint stress images of mice during gait test recorded by camera. H) Representative footprint and I) corresponding footprint area of right hind limb in each group (n = 15; ∗*p* < 0.05, ∗∗*p* < 0.01, ∗∗∗*p* < 0.001). J) Representative 2D and 3D footprint stress images and K) quantitative analysis of footprint stress (n = 3; ∗*p* < 0.05, ∗∗*p* < 0.01).

Restoring muscle function is crucial for muscle injury repair. To further assess functional muscle regeneration, we performed the CatWalk test after four weeks of treatment to record the footprints of mice walking on a transparent runway for gait analysis. Representative gait graphs of mice from each group during the test are shown in Fig. 10A. In the marching graphs, right forepaw (RF) is marked in yellow, right hindpaw (RH) in green, left forepaw (LF) in red, and left hindpaw (LH) in purple. Stride length is an important index for evaluating walking speed and stability, and it can be quantitatively analyzed based on the recorded footprints. Compared with the muscle injury groups treated with the P (5.29 ± 0.61 cm) and PG (5.76 ± 0.62 cm) scaffolds, the stride length of mice in the PGH group increased significantly to 6.39 ± 0.66 cm (Fig. 10C). Additionally, we assessed gait patterns by comparing the base of support (BOS) and the regularity index (RI). BOS measures the distance between the two hindlimbs of the experimental animals, reflecting the support and stability of the animal's feet during walking [66]. The BOS values for the P, PG, and PGH groups were 2.13 ± 0.08, 2.05 ± 0.07, and 1.88 ± 0.10, respectively (Fig. 10D). Compared to the P and PG scaffolds, the PGH scaffold-treated mice exhibited significantly lower BOS, indicating better walking stability. Similarly, RI measures gait coordination and represents the percentage of normal step sequence patterns [66]. Compared to the P scaffold-treated group (77.81 ± 6.12 %), the RI in the PGH scaffold-treated group significantly increased to 94.51 ± 5.56 %, indicating improved gait coordination and functional integrity (Fig. 10E). The representative step-sequence chart of mice in the PGH group showed that their stepping pattern was alternating and very stable (Fig. 10B). Footprint area and stress can reflect how the sole of the animals' feet make contact with the ground. The footprint stress images of mice in each group during the gait test, recorded by the camera, are shown in Fig. 10G. Based on the representative footprint images, the footprint area of the P scaffold-treated group was the smallest, with the toe and sole of the hindpaw connected. In contrast, the footprint area of the PGH scaffold-treated group was the largest, with clear separation between the toe and sole of the hindpaw (Fig. 10H and I). Furthermore, representative 2D and 3D footprint stress images, as well as the quantitative analysis of footprint stress, are provided in Fig. 10J and K. Compared to the P scaffold-treated group, the footprint stress of mice treated with the PG and PGH scaffolds was significantly reduced. These results, along with the footprint area and stress analysis, suggest that mice treated with the PGH scaffold experienced less pain or inflammation. The holding power of the hindpaw in each group was also tested, and there was no significant difference between the P, PG, and PGH scaffold-treated groups (Fig. 10F). The hindpaw holding power reflects muscle function recovery, which may require extended treatment time and muscle rehabilitation training for further improvement. Overall, the gait test results confirmed that the PGH scaffold promoted skeletal muscle regeneration and functional recovery in mice with TA muscle injury.

## 3 Conclusion

In summary, a sandwich-like electrospun fibrous scaffold (PGH) was conceptualized, prepared, and validated *in vitro* and *in vivo* for skeletal muscle regeneration. The present study demonstrated that the optimized, aligned PCL/gelatin fibers can promote C2C12 cell adhesion, cytoskeletal and nuclear remodeling, as well as myogenic differentiation. Due to the sandwich structure design of the fibrous scaffold, the PGH scaffold exhibits excellent tensile strength, elongation at break, and suture retention strength, making it suitable for skeletal muscle regeneration and clinical applications. Notably, exogenous hyaluronic acid significantly enhances the myogenic differentiation of C2C12 cells and facilitates macrophages phenotype transformation, creating a favorable immune and skeletal muscle regeneration niche. Importantly, ultrasound imaging, histological analysis, and transcriptomic profiling showed that muscle injury treated with the sandwich-like PGH scaffold accelerates myogenesis, promotes vascular maturation, manages inflammation, and supports the formation of muscle fibers, ultimately restoring the function of injured muscle. However, the long-term efficacy of the sandwich-like PGH scaffold *in vivo* needs to be further evaluated to fully investigate their degradation behavior and potential chronic inflammatory responses, and to restore muscle function. In addition, the fatigue resistance of the sandwich-like PGH scaffold also need to be further investigated, because the high frequency dynamic muscle contraction will cause the mechanical properties of the scaffold to decay during service. Overall, the sandwich-like electrospun PGH fibrous scaffold can effectively initiate immune regulation and skeletal muscle regeneration, making it a promising therapeutic strategy for muscle injury.

## 4 Experimental section

### 4.1 Materials and reagents

PCL (*M*n = 80∼100 kDa), Gelatin (pharmaceutical grade), hyaluronic acid (HA; *M*_w_ = 800–1500 kDa), Coumarin-6, Coumarin-120, 1,1,1,3,3,3-hexafluoro-2-isopropanol (HFIP), and 2,2,2-trifluoroethanol (TFE) were purchased from Aladdin Co., Ltd. (China). Ethyl alcohol and formic acid (FA, analytical reagent) were purchased from Chengdu Kelong Co., Ltd. (China). DAPI and actin-tracker red-594 were purchased from Beyotime (China). Fluorescent secondary antibodies (ab150077, ab150115), Alexa Fluor® 647 Rat monoclonal to F4/80 antibody (ab204467), Alexa Fluor® 568 Rabbit monoclonal to iNOS antibody (ab209595), rabbit monoclonal to mannose receptor antibody (ab300621), anti-CD31 antibody (ab222783), rabbit recombinant multiclonal to CD68 antibody (ab303565), anti-vinculin antibody (ab129002), anti-myosin heavy chain antibody (ab37484) and anti-laminin 2 alpha antibody (ab259345) were purchased from Abcam (UK). Anti-ACTA2 antibody (orb195993) was purchased from Biorbyt (UK). Anti-Pax7 antibody (Cat#AB528428) was purchased from DSHB laboratory (USA). FITC-linked polyclonal antibody to Hyaluronan Binding Protein 1 (Cat#LAA651Mu81) was purchased from Cloud-Clone Corp. Wuhan (China). APC anti-mouse CD163 antibody (Cat#155305) and FITC anti-mouse F4/80 antibody (Cat#123107) for flow cytometry were purchased from Biolegend (UK). PE Rat anti-mouse CD86 antibody (Cat#561963) was purchased from BD Biosciences (USA). Rhodamine B and horse serum were obtained from Beijing Solarbio Science & Technology Co., Ltd. (China). Dulbecco's modified Eagle medium (DMEM) with high glucose, phosphate-buffered saline (PBS), tyrisin, and fetal bovine serum (FBS) were purchased from Gibco (Thermo Fisher Scientific, USA).

### 4.2 Fabrication of electrospun fibrous scaffolds

To fabricate pristine aligned PCL (Ali) and random PCL (Ran) fibrous scaffolds, 1.3 g of PCL particles were dissolved in 10 mL of TFE. The Ali and Ran scaffolds were then fabricated using an electrospinning device (SS-2535H, Beijing Ucalery Co., Ltd., China), with a cylindrical receiver rotating at speeds of 2000 rpm and 120 rpm, respectively. For preparing aligned gelatin-modified PCL scaffolds, different weight ratios of PCL to gelatin (100:0, 70:30, 50:50, and 30:70, abbreviated as PCL, P7G3, P5G5, and P3G7, respectively) were dissolved in 10 mL of TFE containing 50 μL of FA. The total amount of solute for the PCL, P7G3, P5G5, and P3G7 scaffolds was 1.3 g. Gelatin-modified PCL scaffolds were also fabricated using a cylindrical receiver at a rotational speed of 2000 rpm. After optimizing the gelatin proportion (70:30, P7G3), sandwich-structured fibrous scaffolds containing hyaluronic acid (HA) were prepared. Briefly, the aligned P7G3 fibrous layer was first collected by the cylindrical receiver at 2000 rpm. Then, a random fibrous layer containing hyaluronic acid was collected at 120 rpm, followed by the collection of a second aligned P7G3 fibrous layer at 2000 rpm. The collection time for all three layers of the sandwich-structured fibrous scaffolds was kept the same. The receiving distance between the cylinder and the needle tip (21G) was 12 cm, and the voltage was set to 10 kV. The flow rate of the electrospinning solution was 0.55 mL/h. For fabricating the random fibrous layer of the sandwich-structured scaffolds, 0.91 g of PCL and 0.39 g of gelatin were dissolved in 10 mL of TFE containing 50 μL of FA and used as the outer coaxial needle solution. As mentioned previously, the electrospinning parameters were similar, with the main difference being that the needle was coaxial (25G/18G). The outer flow rate was 0.36 mL/h, and the inner flow rate was 0.18 mL/h. Simultaneously, 0.65 g of PCL and 0.095 g of HA were dissolved in 5 mL of TFE and FA (in an 8:2 vol ratio) and used as the solution for the inner coaxial needle. To simplify the abbreviations, the pristine aligned PCL scaffold is referred to as "P", the aligned P7G3 scaffold as "PG", and the sandwich-structured fibrous scaffold containing HA as "PGH".

### 4.3 Characterization of electrospun scaffolds

The morphology of the scaffolds with different compositions and structures was observed using scanning electron microscopy (SEM; GeminiSEM 300, ZEISS, Germany). The fiber diameters were measured from the SEM images. The Fast Fourier Transform (FFT) spectrogram images, fiber orientation, dispersion, and the alignment quality of fibers (whether aligned or random) were analyzed using the open-source platform Fiji, ImageJ. The wettability of different gelatin compositions was evaluated using a contact angle measurement instrument (JY-82B; Chengde Dingsheng, China). Laser micro-confocal Raman spectroscopy (LabRAM HR Evolution, HORIBA, Japan) was employed to observe the distribution of gelatin in the P7G3 fibers. The laser wavelength was set to 633 nm, and the scanning range for the mapping was 5 × 5 μm. Transmission electron microscopy (TEM; JEM-2100Plus, JEOL, Japan) was used to observe the coaxial structure of the random fibers. The height fluctuations and roughness of the aligned and random fibers were analyzed by atomic force microscopy (AFM; Dimension ICON, Bruker AXS, Malaysia) in tapping mode at a sweep frequency of 1 Hz. The data and images were exported using NanoScope Analysis 3.0 software (Bruker, Malaysia). To evaluate the collection efficiency of aligned fibers and observe the three-layer structure of the PGH scaffolds, fibers labeled with Coumarin-6, Coumarin-120, and Rhodamine B were visualized using stochastic optical reconstruction microscopy (STORM; TiA1-N-STORM, Nikon, Japan). The mechanical properties of the fibrous scaffolds were tested using an electronic universal testing machine (Instron 5967, INSTRON, USA). All tensile specimens were prepared in a standard dumbbell shape, with a total length of 50 mm and parallel segment width of 4 mm. The stretching speed was set to 15 mm/min. For suture retention strength testing, the specimen was cut into a rectangle, 3 mm wide and 1 mm thick. A 5-0 polyester suture was passed through two-thirds of the specimen, 2 mm from the upper short edge. The suture was fixed at the upper clamp, and the specimen was fixed at the lower clamp. The specimen was stretched at a speed of 10 mm/min until the suture tore through the specimen.

### 4.4 Cell and animal

C2C12 cells were obtained from Warner Bio (Wuhan) Co., Ltd. (China) and cultured in DMEM (Dulbecco's Modified Eagle Medium) supplemented with 10 % fetal bovine serum (FBS) and 1 % penicillin-streptomycin (MP Biomedicals, USA). For myogenic differentiation, the medium was modified to be FBS-free and supplemented with 2 % horse serum. NIH3T3 and RAW264.7 cells were cultured in DMEM containing 10 % FBS and 1 % penicillin-streptomycin. The FBS used for culturing RAW264.7 cells was heat-inactivated by incubating at 56 °C for 2 h. All cells were cultured at 37 °C in a 5 % CO_2_ incubator. C57BL/6 mice (female, 6–8 weeks old) were obtained from Byrness Weil Biotech Ltd. (China). All animal experiments were conducted in accordance with the guidelines approved by the Institutional Animal Care and Use Committee (IACUC) of Sichuan University (Approval No. 20240301196). Additionally, all scaffolds used in cell and animal experiments were sterilized using UV light in a biological safety cabinet (1300 Series A2, Thermo Scientific, USA).

### 4.5 *In vitro* cell experiments

C2C12 and NIH3T3 cells were seeded at 5 × 10^3^ cells per well in 48-well plates containing aligned or random PCL scaffolds. After 3 days of incubation, the cells were fixed with 4 % paraformaldehyde (PFA) and stained with DAPI and Actin-Tracker. The cell morphology was then observed using stochastic optical reconstruction microscopy (STORM), and the cytoskeletal arrangement and the aspect ratio of the fitted ellipse of the nucleus were analyzed using ImageJ software. Additionally, transcriptome sequencing (Shanghai Biotechnology Corporation, China) was performed to examine the expression of relevant genes. The sequencing was carried out on an Illumina NovaSeq 6000 platform using the PE150 sequencing mode. C2C12 cells were seeded on aligned and random PCL scaffolds and cultured for 5 days in myogenic differentiation medium. The expression of MHC was assessed via fluorescence staining.

C2C12 cells were seeded at 2 × 10^4^ cells per well in 24-well plates containing P, PG, or PGH scaffolds and cultured for 3 days. Vinculin expression was visualized using fluorescence staining, and the cell morphology was observed using scanning electron microscopy (SEM). Additionally, the expression of MHC protein was measured after 3 days of myogenic induction culture on P, PG, and PGH scaffolds. The mean fluorescence intensity and aspect ratio were analyzed using ImageJ software. After 3 days of myogenic differentiation, the gene expression levels of myogenic markers (*Myf5*, *MyoD*, and *MyoG*) were evaluated by qPCR, and the primer sequences are provided in Table S1. Gene expression fold changes were calculated using the 2^−ΔΔCT^ method.

RAW264.7 cells were seeded at 1 × 10^4^ cells per well in 96-well plates and cultured with the extract from P, PG, or PGH scaffolds. After 24 h, the cells were stained with anti-F4/80, anti-iNOS, and anti-CD206 antibodies. The fluorescence intensity of these markers was measured using high-content screening (HCS) with an Opera Phenix Plus system (PerkinElmer, USA), and the data were analyzed with Harmony software (PerkinElmer, USA). For flow cytometry analysis, RAW264.7 cells were seeded at 1 × 10^5^ cells per well in the lower chamber of 12-well plates, with the different scaffolds placed in the upper chamber. After 24 h of co-culture, cells were labeled with anti-F4/80, anti-CD86, and anti-CD163 antibodies, and analyzed by flow cytometry (FACSAria III, BD, USA).

### 4.6 Tibialis anterior muscle defect model

C57BL/6 mice were continuously anesthetized with isoflurane through respiratory inhalation. The surgical area was disinfected with iodophor. A surgical blade was used to make an 8 mm skin incision, and blunt dissection was performed to expose the tibialis anterior (TA) muscle. The TA muscle was lifted with blunt forceps, and approximately 50 % of its volume (around 4 mm long, 3 mm wide, and 0.8 mm deep) was resected using the blade. Electrospun fibrous scaffolds (P, PG, and PGH) were then implanted onto the TA muscle to completely cover the defect site. A 5-0 surgical suture was used to secure both ends of the scaffold to the muscle, and the skin incision was closed. Both legs of each mouse underwent the same surgical procedure and scaffold implantation. In the control group, after the muscle defect was created, the incision was simply rinsed with normal saline and sutured without scaffold implantation. Euthanasia was performed at a predetermined time point following instrumental assessments, and muscle samples were collected and fixed in 4 % PFA for subsequent histological analysis.

### 4.7 Imageological analysis

For ultrasound imaging, the animals were anesthetized with isoflurane via respiratory inhalation and fixed on a stage. Hair in and around the surgical area was removed using a hair removal cream. After applying a layer of medical ultrasound coupling gel over the tibialis anterior (TA) muscle, high-frequency ultrasound imaging was performed in the sagittal plane using an ultrasound probe. Ultrasound data were acquired with the US/PA imaging system (Vevo 3100, FUJIFILM VisualSonics, USA), using the MX550D probe at a frequency of 40 MHz. The B-mode ultrasound imaging module was used, and images were captured from the superficial abdominal tissue. The ultrasound images were exported using Vevo LAB software and analyzed semi-quantitatively with ImageJ software.

### 4.8 Histological analysis

After preparing paraffin sections, the sections were stained with H&E and Masson. The degree of muscle repair, myofiber diameter, and the number of myofibers in the muscle defect area were assessed using ImageJ software. To evaluate the local immune microenvironment, CD68 was used as a marker for macrophages in immunohistochemistry (IHC) staining. Macrophage subtypes were further distinguished using immunofluorescence (IF) staining for CD206 and iNOS, respectively. Additionally, IF staining for CD31 and ACTA2 was performed to visualize neovascularization in the defect area. The number of CD68^+^, CD206^+^, and iNOS^+^ macrophages, as well as vascular density, were evaluated using ImageJ software. Furthermore, IF staining for muscle-specific markers (MHC, Laminin, and Pax7) and hyaluronic acid-binding proteins (HABP) was conducted to assess skeletal muscle regeneration. The expression levels of these proteins were analyzed using ImageJ software. Primary antibodies were used at the recommended concentrations, and all staining procedures were performed by incubating the sections overnight at 4 °C.

### 4.9 Transcriptome sequencing

Transcriptome sequencing was performed two weeks after surgery. Samples for bulk RNA sequencing (Bulk RNA-seq) were collected from fresh tibialis anterior (TA) muscle of the PG and PGH groups. The samples were immediately stored in liquid nitrogen after being separated from the animals. Total RNA was extracted using the MJzol Animal RNA Isolation Kit (Majorivd). RNA purification was performed with the RNAClean XP Kit (Beckman Coulter) and treated with the RNase-Free DNase Set (QIAGEN) to remove any contaminating DNA. Sequencing was conducted on the Illumina NovaSeq6000 platform using the PE150 (paired-end 150 bp) sequencing mode. Differential gene expression analysis was carried out using the bioinformatics cloud system provided by Shanghai Biotechnology Corporation (https://tools.shbio.com/). Gene Ontology (GO) and Kyoto Encyclopedia of Genes and Genomes (KEGG) terms with an adjusted p-value (P_adj) < 0.05 were considered significantly enriched.

### 4.10 Gait analysis and grip test

The changes in the mice's gait were detected using the VisuGait analysis system (XR-FP101, Shanghai Xinsoft Information Technology Co., Ltd., China) designed for rodents. Briefly, the mice were acclimated and trained on the track in the facility for 3 days before the formal experiment. A high-speed camera positioned below the track recorded the mice's movements in their natural state without interruption. Motor ability was evaluated by analyzing the footprints and categorizing the limb movements interactively (right forepaw, RF; right hindpaw, RH; left forepaw, LF; left hindpaw, LH). Stride length refers to the distance between two successive strides of the same paw. The base of support (BOS) of the hind limbs is the lateral distance between the two hind paws. The regularity index (RI) measures interlimb coordination. Step order is an important index used to evaluate the coordination and functional integrity of the animal's gait. Footprint area and footprint pressure were calculated based on the walking video. Progress maps, step order maps, as well as 2D and 3D footprint pressure diagrams were generated using the VisuGait animal visual analysis system software. In addition, the grip strength of the mice's hind limbs was measured using a grip meter (ZS-ZL, Beijing ZS Dichuang Technology Co., Ltd., China).

### 4.11 Statistical analysis

All graphs in this article were generated using Origin 9.0 (OriginLab Corporation, USA) and GraphPad Prism 8.0.1 (GraphPad Software Inc., USA). Data are presented as the mean ± standard deviation (SD). Statistical analysis was performed using GraphPad InStat 3.05 (GraphPad Software Inc., USA). One-way analysis of variance (ANOVA) with Tukey's post hoc test was used to compare more than two groups, while an unpaired *t*-test was used for comparisons between two groups. A p-value of less than 0.05 was considered statistically significant (∗*p* < 0.05, ∗∗*p* < 0.01, ∗∗∗*p* < 0.001, ∗∗∗∗*p* < 0.0001).

## CRediT authorship contribution statement

**Shue Jin:** Writing – original draft, Methodology, Investigation, Funding acquisition, Formal analysis, Data curation, Conceptualization. **Yongrui Cai:** Visualization, Methodology, Investigation. **Yaxing Li:** Methodology, Investigation. **Jing Wen:** Validation, Software, Investigation, Formal analysis, Data curation. **Xiaoxue Fu:** Visualization, Resources, Methodology, Investigation. **Ping Song:** Methodology, Investigation. **Pengyu Lu:** Methodology, Investigation. **Anjing Chen:** Project administration, Investigation. **Zeyu Luo:** Resources, Methodology. **Weinan Zeng:** Writing – review & editing, Supervision, Resources, Project administration. **Jidong Li:** Writing – review & editing, Resources. **Zongke Zhou:** Writing – review & editing, Supervision, Resources, Project administration, Funding acquisition.

## Data availability statement

All data need to support the conclusion of this study are presented in the paper and available from the corresponding author upon reasonable request.

## Ethics approval and consent to participate

All animal experiments were conducted in accordance with the guidelines approved by the Institutional Animal Care and Use Committee (IACUC) of Sichuan University (Approval No. 20240301196).

## Declaration of competing interest

The authors declare that they have no competing interests.
