# Supplementary material for: A sandwich-like nanofibrous scaffold with macrophage phenotype transformation and myogenic differentiation for skeletal muscle regeneration
Source: Bioact Mater. 2025 May 13;51:211–30. doi: 10.1016/j.bioactmat.2025.05.008 (PMC12141875; doi:10.1016/j.bioactmat.2025.05.008)
Supplement: Multimedia component 1 [file mmc1.docx]

**A Sandwich-like Nanofibrous Scaffold with Macrophage Phenotype Transformation and Myogenic Differentiation for Muscle Regeneration**

**Table S1. The primer sequence for qPCR involved in this study**

| Gene | Forward primer | Reverse primer |
| --- | --- | --- |
| *Myf5* | TCAGGAATGCCATCCGCTAC | ACAGGGCTGTTACATTCAGGCAT |
| *MyoD* | ATGGCTACGACACCGCCTACTA | CACCTGATAAATCGCATTGGG |
| *MyoG* | AGCGGCTGCCTAAAGTGGA | ATTGTGGGCGTCTGTAGGGT |
| *Myh1* | GCGAATCGAGGCTCAGAACAA | GTAGTTCCGCCTTCGGTCTTG |
| *Myl2* | ATCGACAAGAATGACCTAAGGGA | ATTTTTCACGTTCACTCGTCCT |
| *Myom1* | TCATCCCGGCATTCTCAAGG | GTCCGTGGGAATAGCCGTAAT |
| *Cxcr2* | CCTCAAACGGGATGTATT | GCTCTGTCACCGATGTCT |
| *Cxcl2* | CGCTGTCAATGCCTGAAG | GGCGTCACACTCAAGCTCT |
| *Ccl3* | TGTACCATGACACTCTGCAAC | CAACGATGAATTGGCGTGGAA |
| *IL1β* | GAAATGCCACCTTTTGACAGTG | TGGATGCTCTCATCAGGACAG |


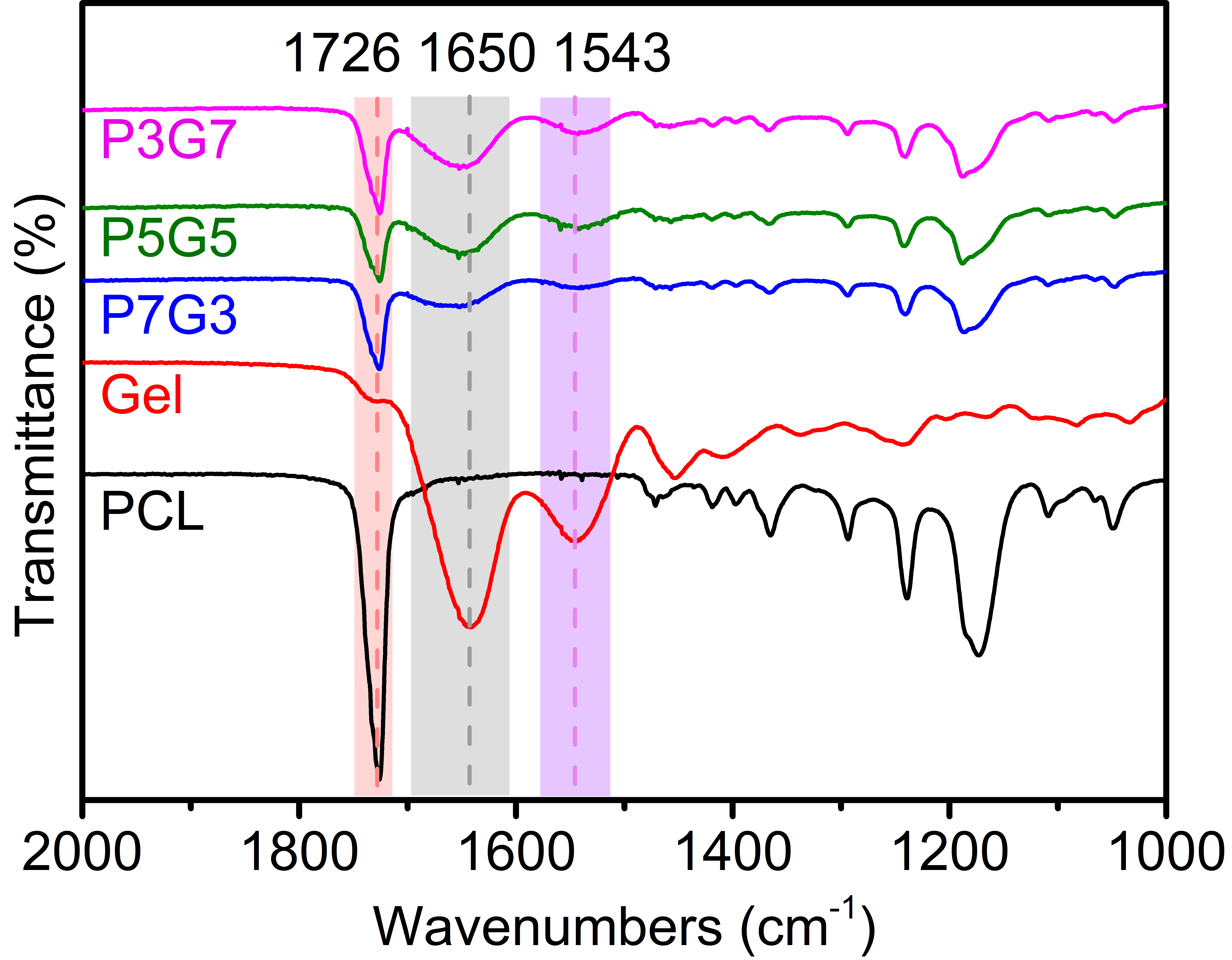


**Figure S1.** FTIR spectra of fibrous scaffolds with different content of gelatin.


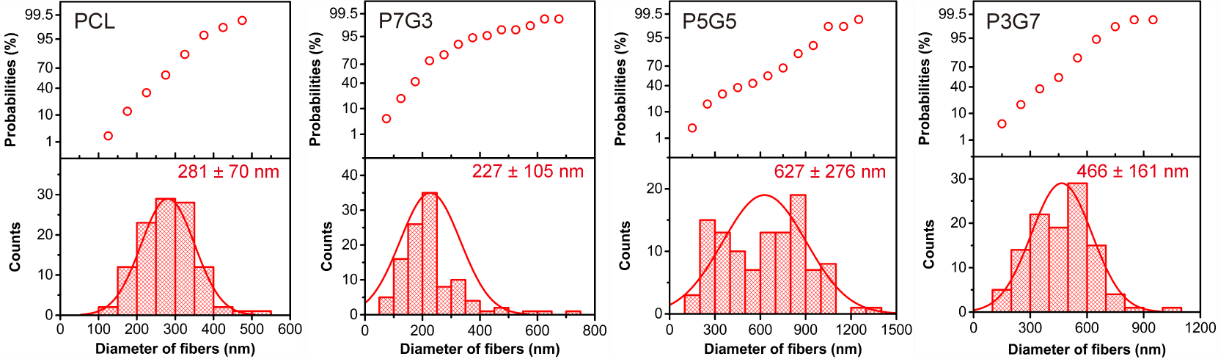


**Figure S2.** The fiber diameter distribution and probabilities of PCL, P7G3, P5G5, and P3G7 scaffolds.


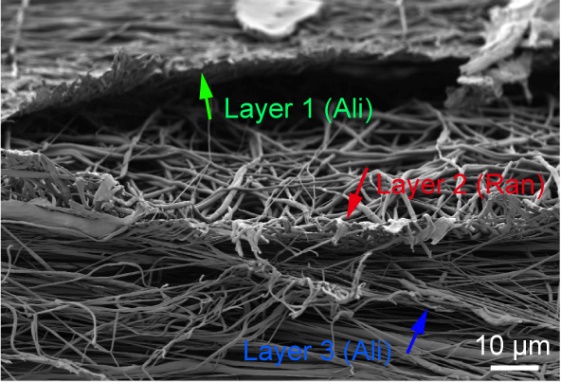


**Figure S3.** The SEM image of cross section of sandwich-like three layers fibrous scaffold.


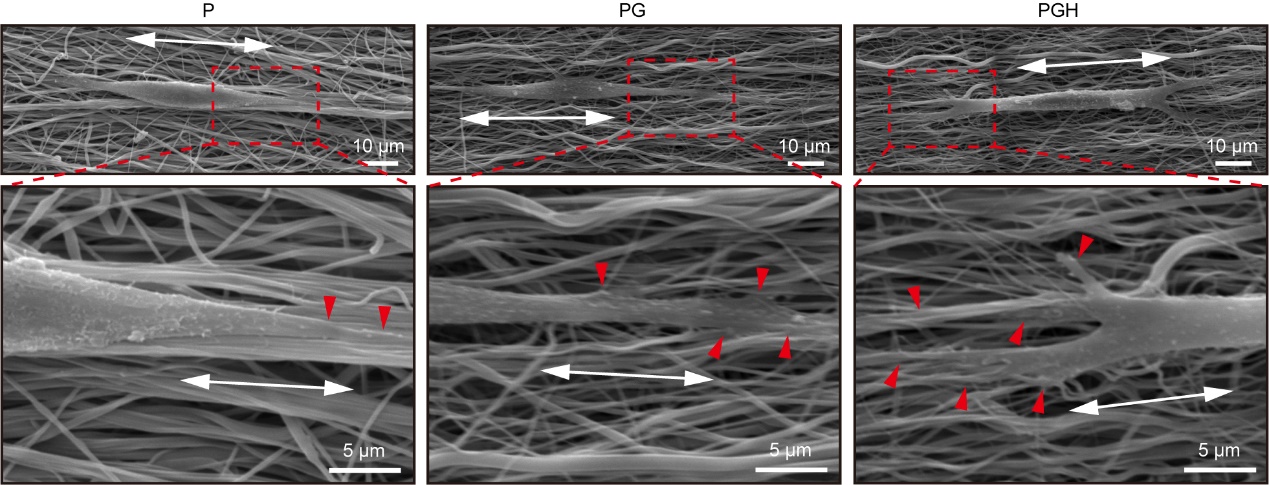


**Figure S4.** The SEM images of C2C12 cells culturing on P, PG and PGH scaffolds for 3 days. The white arrows indicate the direction of the cytoskeleton, and the red arrows indicate the pseudopods where the cells adhere to the fibers.


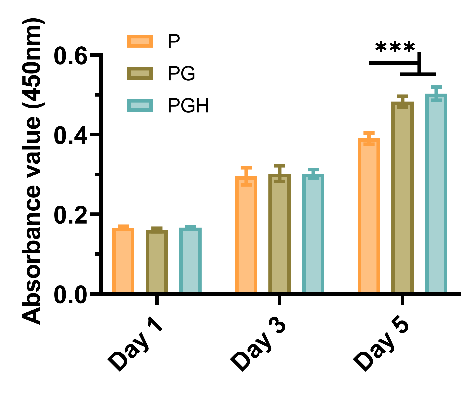


**Figure S5.** The proliferation of C2C12 cells on P, PG and PGH scaffolds. (n = 5; ****p* < 0.001).


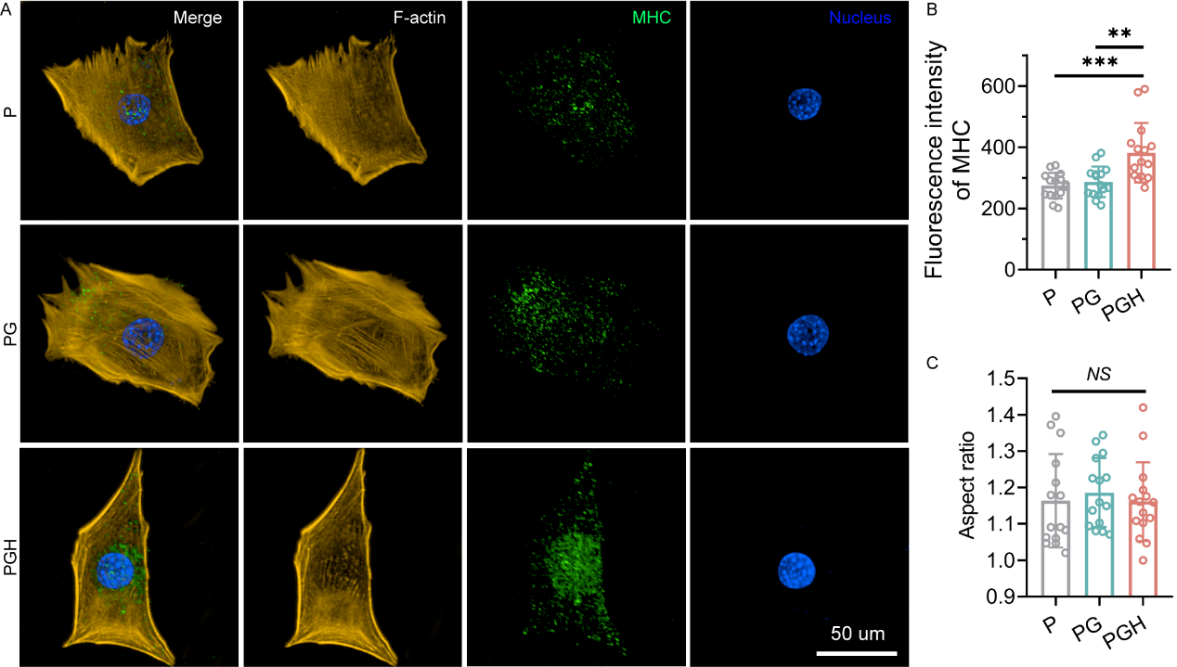


**Figure S6.** A) The fluorescence images of C2C12 cells culturing with extract liquid of P, PG, and PGH scaffolds after 3 days. B) The MHC expression level of C2C12 cells on P, PG, and PGH group. C) The aspect ratio of nucleus in P, PG, and PGH group. (n = 15; ***p* < 0.01, ****p* < 0.001).


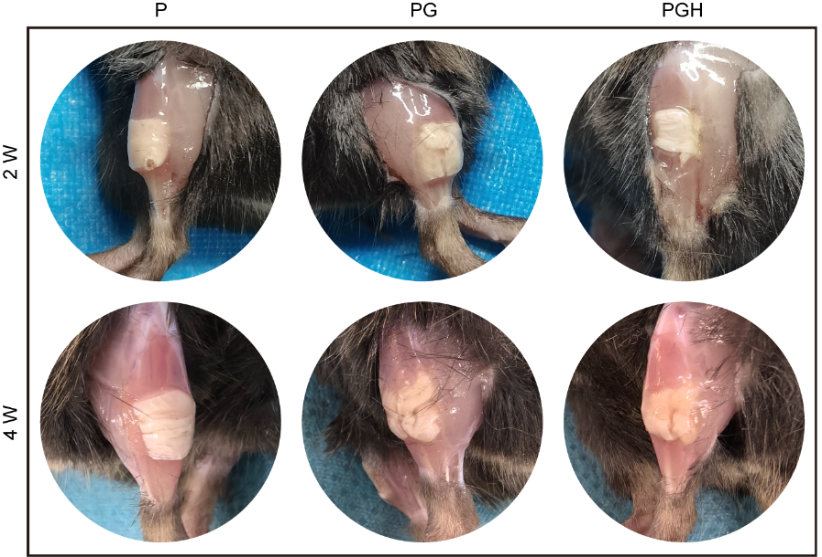


**Figure S7**. Macroscopic photos of three scaffolds at the time of sample collection.


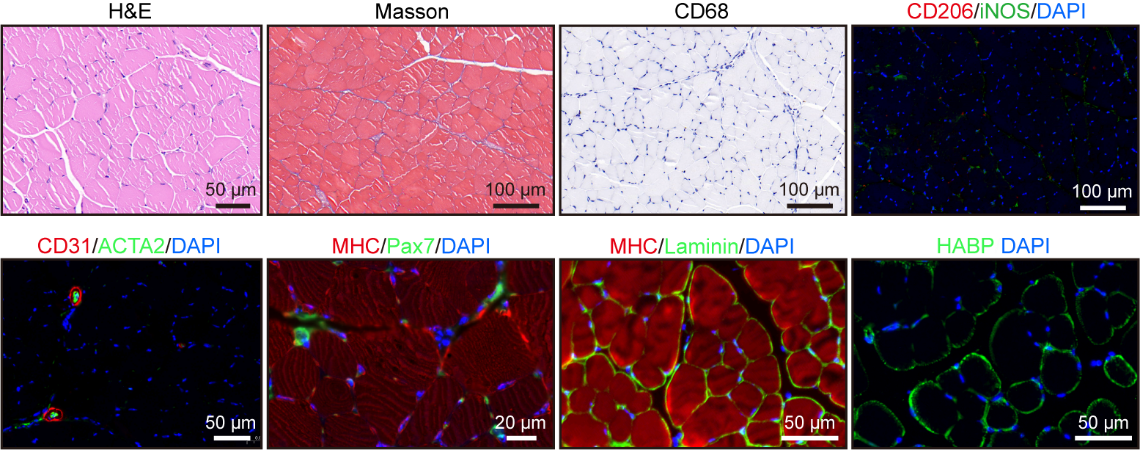


**Figure S8.** Histological staining of a normal muscle sample.
